# Dissociable Mechanisms Underlie Differences Between Memory and Metamemory in Older Adults: The Differentiating Role of Anxiety and Depression Symptoms

**DOI:** 10.1002/hipo.70100

**Published:** 2026-04-19

**Authors:** Jennifer L. Crawford, Alex A. Adornato, Johanna Matulonis, Xi Chen, Jacob M. Hooker, Anne S. Berry

**Affiliations:** ^1^ Department of Psychology Brandeis University Waltham Massachusetts USA; ^2^ Department of Psychology Stony Brook University Stony Brook New York USA; ^3^ Martinos Center for Biomedical Imaging Massachusetts General Hospital Charlestown Massachusetts USA

**Keywords:** gray matter volume, hippocampus, neuropsychiatric symptoms, self‐reported memory, tau pathology

## Abstract

The ability to remember (i.e., memory ability) and to accurately discern memory function (i.e., metamemory) are both important facets of cognition. In the present study, we examined the shared and distinct sources of variance across memory ability and metamemory using psychometrically validated measures of memory ability, metamemory, and anxiety and depression symptoms in conjunction with multimodal imaging (i.e., structural MRI, tau PET) in a sample of cognitively normal older adults (*N* = 72). Replicating a growing body of work, we found that metamemory was more tightly linked to anxiety and depression symptoms relative to objective measures of memory ability. Our results also revealed that the hippocampus was a critical locus of both memory ability and metamemory—hippocampal volume was positively associated with memory ability, but not metamemory, whereas increased hippocampal tau pathology exacerbated the negative effect of anxiety and depression symptoms on metamemory. Importantly, we also found that after controlling for anxiety and depression symptoms and tau burden, there was a positive association between memory ability and metamemory. Our findings also demonstrated the importance of assessing different facets of metamemory; self‐reported memory contentment and ability, but not strategy use, showed the strongest relationships with both anxiety and depression symptoms and hippocampal tau burden. Together, these results suggest that both shared and distinct mechanisms underlie memory ability and metamemory processes in older adults. Chiefly, this work highlights the potential of metamemory measures as sensitive tools to understand affective processes that occur in both healthy and pathological aging, independent of memory ability.

## 1 Introduction

Memory is a focal point of many studies of aging and Alzheimer's disease (AD). Although many investigations using neuropsychological assessments have shown that memory ability tends to decline with age (Nyberg and Pudas 2019; Reuter‐Lorenz and Park 2014), and is further impacted by AD pathology (Aschenbrenner et al. 2018; Chen et al. 2025; Maass et al. 2018; Pezzoli et al. 2025), assessments indexing metamemory—self‐reported assessments measuring the knowledge and awareness of one's own memory processes—do not always closely correspond to experimentally‐assessed measures of memory ability (Hertzog and Pearman 2013). Indeed, meta‐analyses have found that how participants perform on memory assessments was only weakly related to how they rated their own memory ability (i.e., metamemory) (Beaudoin and Desrichard 2011; Crumley et al. 2014). Though there is an apparent disconnect between objective and subjective measures of memory ability in aging, there is growing consensus that subjective cognitive complaints are clinically relevant. Namely, subjective cognitive decline is associated with increased likelihood of developing dementia, in addition to having the potential to dramatically affect well‐being (Jessen et al. 2014; Rabin et al. 2017). As such, understanding the mechanisms underlying distinct memory‐related processes in older adults, such as memory ability and metamemory, is important to be able to identify individuals at greater risk for developing dementia and to develop potential strategies to mitigate risk.

Affective function has been identified as a potential confounding variable when examining the linkages between memory ability and metamemory in older adults. Measures of both anxiety and depression symptoms consistently demonstrate robust relationships with self‐reported metamemory (Hill et al. 2016). In kind, multiple studies have found that measures of metamemory were more strongly associated with anxiety and depression symptoms than with measures of memory ability (Gustavson et al. 2021; Pearman et al. 2014; Tang et al. 2025; Zlatar et al. 2018). Depression symptoms also have been shown to moderate the effect size of the relationship between memory ability and metamemory; studies with greater numbers of participants with elevated levels of depression symptoms tended to observe smaller correlations between memory ability and metamemory (Crumley et al. 2014). Further, a coordinated analysis across four longitudinal datasets revealed that depression symptoms partially mediated the association between memory ability and metamemory (Hill et al. 2021), which suggests that older adults with worse self‐reported memory and elevated levels of depression symptoms are more likely to have greater objectively assessed memory deficits. Taken together, these findings highlight the importance of including affective measures in studies of memory function in older adults to more precisely understand the relationships between memory ability and metamemory and their underlying mechanisms.

Investigations into the neural substrates of memory ability and metamemory reveal both shared and distinct associations with brain structure. For example, there is growing evidence that estimates of gray matter volume in the medial temporal lobe are important for both memory ability and metamemory in older adults. Namely, gray matter volume in both the entorhinal cortex and hippocampus has been shown to be positively associated with measures of memory ability both cross‐sectionally and longitudinally (Aghjayan et al. 2025; Maass et al. 2018; Ottoy et al. 2019). In contrast, although some studies have observed associations between cross‐sectional estimates of hippocampal volume and metamemory (Hafkemeijer et al. 2013), other work has failed to observe this association after controlling for memory ability and anxiety and depression symptoms (Amariglio et al. 2015).

In parallel, investigations that have assessed AD pathology and its associations with memory ability and metamemory have also shown clear dissociations across the two memory‐related constructs. For example, studies examining the relationships between memory ability and AD pathology have found that tau pathology, relative to beta‐amyloid burden, is particularly important for our understanding of declines in memory ability in older adults (Chen et al. 2024, 2025; Fonseca et al. 2024; Pezzoli et al. 2025). On the other hand, subjective memory measures indexing metamemory show associations with both beta‐amyloid and tau pathology in which individuals with worse subjective memory have higher levels of pathological burden (Buckley et al. 2017; La Joie et al. 2016; Perrotin et al. 2012, 2017), which might suggest a more generalized AD pathological mechanism underlying metacognitive deficits, relative to measures of memory ability. Nevertheless, it is important to note that early tau PET tracers, including those used in the prior work assessing the relationships between tau burden and metamemory (Buckley et al. 2017), were unable to reliably quantify hippocampal tau burden (Betthauser et al. 2019). As such, there have been no studies to date that have examined the extent to which measures of metamemory are associated with tau pathology in the hippocampus, which represents a significant gap in our knowledge, given the importance of the hippocampus to memory processes in aging and AD.

Understanding the relationships between memory ability and metamemory, and their underlying mechanisms, also requires careful consideration of how self‐reported measures of metamemory are assessed. Although assessments of memory ability differ across studies, a far greater degree of heterogeneity has been noted in the literature on assessments of metamemory, and related processes, both regarding the types of measures used and the number of items used to assess metamemory. For example, a recent analysis across 19 large‐scale studies found that there was little overlap of the measures used to assess subjective estimates of cognition, with 75% of the self‐report measures used by only one study (Rabin et al. 2015). Moreover, this same analysis showed that the number of items used to assess subjective cognition varied widely—over a third of the instruments only contained a handful of items, with some only including a single item (Rabin et al. 2015). Using only one item, or a limited number of items, to assess metamemory not only risks failing to reliably assess this construct (Hayes et al. 2017), but it also eliminates the opportunity to understand different facets of metamemory in relation to objective assessments of memory ability. Multiple, psychometrically validated, questionnaires have shown that metamemory is a multifaceted construct; these instruments contain different sub‐scales indexing memory ability, capacity, contentment, strategy use, perceived change, and other similar constructs (Gilewski et al. 1990; Hertzog et al. 1987; Troyer and Rich 2002). Indeed, studies using these questionnaires have found that only specific facets of metamemory (e.g., capacity, perceived change) are related to longitudinal changes in objectively assessed memory ability (Chen et al. 2019; Zimprich and Kurtz 2015). Such findings highlight the importance of using measures of metamemory that can reliably assess multiple domains of this construct to rigorously understand its linkages to objective assessments of memory ability and other relevant variables (e.g., affective function, gray matter volume, AD pathology) in older adults.

The current study investigated the extent to which assessments of memory ability and metamemory were associated with each other and further examined the contribution of anxiety and depression symptoms, gray matter volume, and tau pathology towards these constructs. We also separately examined how distinct facets of metamemory—contentment, ability, and strategy use—are associated with objective assessments of memory ability and other relevant variables (e.g., anxiety and depression symptoms, gray matter value, tau burden). In line with prior work, we hypothesized that assessments of memory ability and metamemory would only be weakly associated with each other and that metamemory would show stronger associations with anxiety and depression symptoms than with objective assessments of memory ability. Further, we predicted that gray matter volume in the entorhinal cortex and hippocampus would have stronger associations with memory ability, but not metamemory, whereas tau pathology in the entorhinal cortex and hippocampus would be independently associated with both memory ability and metamemory.

## 2 Materials and Methods

### 2.1 Participants

Participants were adults ages 60 and older recruited from the greater Boston metropolitan area through advertisements, direct mail, and research participant registries. Inclusion criteria for participation included English language proficiency, no lifetime history of neurological disorders, a score ≥ 26 on the Mini Mental State Exam, no history of claustrophobia, no metallic objects or devices in the body deemed non‐compatible with MRI (e.g., pacemakers or other implanted cardiac devices), and no routine exposure to radiation. Individuals were not eligible to complete this study if they were currently taking psychotropic medication (or had taken any psychotropic medications in the past 6 months). A history of psychiatric illness was not exclusionary for the present study. All experimental procedures were approved by the Brandeis University Institutional Review Board and the Partners Human Research Committee of Massachusetts General Hospital (MGH) prior to data collection. Participants provided informed consent and were compensated for all study procedures. The sample consisted of 72 participants (39 females, 33 males; *M*_age_ = 69.5; SD_age_ = 5.5; 6 Asian, 7 Black or African American, 58 White, 1 more than one race; 0 Hispanic or Latinx; see Table 1).

**TABLE 1:** Descriptive information for the study sample.

Dimension	Mean (SD)
Age	69.63 (5.42)
MMSE	29.14 (1.03)
GDS	2.87 (3.22)
MASQ: Anhedonic Depression	25.31 (6.84)
MASQ: Anxious Arousal	11.85 (1.89)
MASQ: General Distress	14.18 (4.06)
PSWQ	35.27 (12.29)
Entorhinal Tau (SUVR)	1.37 (0.63)
Hippocampal Tau (SUVR)	0.96 (0.16)
Sex	Percentage
Female	54.17
Male	45.83
Race	Percentage
Asian or Pacific Islander	8.33
Black	8.33
White	81.94
More than One Race	1.39
Ethnicity	Percentage
Hispanic	0
Not Hispanic	100

Abbreviations: GDS, Geriatric Depression Scale; MASQ, Mood and Anxiety Symptom Questionnaire; MMSE, Mini Mental State Exam; PSWQ, Penn State Worry Questionnaire.

### 2.2 Behavioral Assessments

To examine the relationships between measures of memory ability and metamemory we used assessments taken from the Brandeis Aging Brain Study. The Brandeis Aging Brain Study tests participants on an extensive neuropsychological battery containing experimental measures of learning and memory, executive function, and crystallized cognitive ability. Moreover, participants complete an extensive collection of self‐report questionnaires assessing a broad array of constructs including cognitive ability, anxiety and depression symptoms, activity participation, and sleep at each study time point. In the current study, we used experimental assessments and self‐report questionnaires measuring cognition in the domain of memory. As such, we had objective assessments of memory ability and self‐reports of metamemory for each participant. Moreover, these assessments were accompanied by self‐report questionnaires measuring anxiety and depression symptoms to understand the linkages between these constructs. All data used in the current study were taken from participants' initial behavioral assessment, which were collected from December 2019 through October 2024. Data and code used for analyses can be found on the Open Science Framework: https://osf.io/6et32.

#### 2.2.1 Objective Cognitive Assessments

All objective cognitive assessments were performed in the laboratory during the same testing session. All tasks were performed in a fixed order across participants. Descriptive summaries for all measures described below can be found in the Supplement. Memory ability was assessed in using two different tasks: the California Verbal Learning Test (CVLT) (Woods et al. 2006) and a visual reproduction task (Wechsler 1997). The structure of each task is similar in that participants' memory performance is assessed across both long and short delays. As such, the free recall scores across both short and long delays were used in the current study as an index of memory ability, consistent with prior work examining the behavioral and neurobiological correlates of memory function in aging and AD (Chen et al. 2020, 2024, 2025; Tang et al. 2025).

#### 2.2.2 Self‐Report Measures

Self‐report questionnaires were used to assess subjective cognitive ability (i.e., metamemory) and anxiety and depression symptoms. All questionnaires were completed at the same time for all participants and were completed prior to cognitive testing. If an item response was missing from a participant's questionnaire, we imputed the response using the median score for the particular scale from which the response was missing; if over 20% of the items were missing for any given scale, missing items were not imputed, and the participant was not included in the present analysis due to missingness. A summary of imputation and a general descriptive summary for each scale used in the current study can be found in the Supplement. To assess metamemory, we asked participants to complete the Multifactorial Memory Questionnaire (MMQ) (Troyer and Rich 2002). The MMQ has three sub‐scales that index participants' general perceptions about their memory ability (contentment score), how they think their memory functions across different daily life situations (ability score), and utilization of memory strategies across daily life contexts (strategy score); all scores were used to assess self‐perception of memory (i.e., metamemory).

Anxiety and depression symptoms were measured using the combination of the Geriatric Depression Scale (Yesavage et al. 1982) (GDS), the 30‐item Mood and Anxiety Symptom Questionnaire (Wardenaar et al. 2010) (MASQ), and the Penn State Worry Questionnaire (PSWQ) (Meyer et al. 1990). The GDS contains a single scale (GDS score), which captures depressive symptoms, with higher values indicating higher depressive symptoms. Further, the MASQ has three subscales: the anxious arousal scale measures levels of anxiety and somatization, the anhedonic depression score reflects levels of depression‐like symptoms, and the general distress score indexes levels of general psychological distress, depression, anxiety, and somatization. Across all three scales of the MASQ, higher scores indicate greater levels of symptoms or distress. Finally, the PSWQ has only one scale, with higher scores reflecting higher levels of worry. All of the aforementioned scales were included in our composite measure of anxiety and depression symptoms.

### 2.3 Neuroimaging Acquisition and Processing

#### 2.3.1 MRI Acquisition and Processing

As a part of this study, all participants also completed a simultaneous PET‐MRI scan. Scanning was performed on a Siemens 3 T Biograph mMR, capable of simultaneous PET‐MRI acquisition. A T1‐weighted MPRAGE scan was acquired for each participant (TR = 2530 ms, TE = 1.69 ms, Flip angle = 7 degrees, 192 slices, slice thickness = 1.0 mm, FOV = 256 × 256 mm). Results included in this manuscript come from preprocessing performed using *fMRIPrep* 22.0.2 ((Esteban et al. 2019); RRID:SCR_016216), which is based on *Nipype* 1.8.5 ((Gorgolewski et al. 2011); RRID:SCR_002502). The following paragraphs describe the key stages of fMRIPrep preprocessing.

The T1‐weighted (T1w) image was corrected for intensity non‐uniformity (INU) with N4BiasFieldCorrection (Tustison et al. 2010), distributed with ANTs 2.3.3 ((Avants et al. 2008); RRID:SCR_004757), and was used as T1w‐reference throughout the workflow. The T1w‐reference was then skull‐stripped with a *Nipype* implementation of the antsBrainExtraction.sh workflow (from ANTs), using OASIS30ANTs as target template. Brain tissue segmentation of cerebrospinal fluid (CSF), white‐matter (WM) and gray‐matter (GM) was performed on the brain‐extracted T1w using fast (FSL 6.0.5.1, RRID:SCR_002823; (Zhang et al. 2001)). Brain surfaces were reconstructed using ‘recon‐all’ (FreeSurfer 7.2.0, RRID:SCR_001847, @fs_reconall), and the brain mask estimated previously was refined with a custom variation of the method to reconcile ANTs‐derived and FreeSurfer‐derived segmentations of the cortical gray matter of Mindboggle (RRID:SCR_002438, @mindboggle). Volume‐based spatial normalization to one standard space (MNI152NLin2009cAsym) was performed through nonlinear registration with antsRegistration (ANTs 2.3.3), using brain‐extracted versions of both T1w reference and the T1w template. The following template was selected for spatial normalization: *ICBM 152 Nonlinear Asymmetrical template version 2009c* [(Fonov et al. 2009); RRID:SCR_008796; TemplateFlow ID: MNI152NLin2009cAsym].

Both the entorhinal cortex and hippocampus were used as regions of interest in the current study. These regions of interest were defined using the Desikan–Killiany atlas included as the default parcellation within FreeSurfer (Desikan et al. 2006; Fischl 2012); see Supplement for region of interest visualizations. Gray matter volumes were adjusted for total intracranial volume (ICV) using a covariance approach (Buckner et al. 2004) and summed across hemispheres as no a priori effects of hemisphere were expected.

#### 2.3.2 Tau PET Acquisition and Processing

For the [^18^F]MK‐6240 tau PET scan, participants received approximately 5 mCi of tracer (5.28 ± 0.37) and were scanned 90–110 min post‐injection (4 × 5‐min frames) during the same scanning session in which the MRI data were acquired. Standardized uptake value ratio (SUVR) images for MK‐6240 were created by normalizing mean tracer uptake to the inferior cerebellar gray matter reference region (Baker et al. 2017; Harrison et al. 2023). Geometric transfer matrix partial volume correction (PVC) on FreeSurfer‐derived ROIs was applied to account for partial volume effects (Rousset et al. 1998). The entorhinal cortex and hippocampal ROIs, defined by the Desikan‐Killiany atlas, were specifically chosen for the present study because these regions are some of the earliest sites of tau pathology in AD and are known to be important for memory function and metamemory (Braak et al. 2011; Braak and Braak 1995; Braak and Tredici 2015; Buckley et al. 2017; Rabin et al. 2017; Rugg and Vilberg 2013). Unlike first generation tau‐PET tracers, MK‐6240 shows limited off‐target binding in nearby choroid plexus, ensuring an accurate estimation of hippocampal tau burden (Betthauser et al. 2019); see Supplement for visualization of raw PET images with ROI overlay and group averaged SUVR map.

### 2.4 Analysis

To facilitate comparisons across multiple tasks and scales measuring cognition and anxiety and depression symptoms, we created composite scores indexing each construct. As such, three different composites were calculated: objectively assessed memory ability, metamemory, and anxiety and depression symptoms. For each composite, we summed the *z*‐scores across all distinct scores from each instrument. Correlations between the individual scores used to create each composite and the distributions for each composite score can be found in the Supplement.

The relationships between assessments of memory ability and metamemory, along with anxiety and depression symptoms, gray matter volume, and tau pathology were all quantified using linear models. Estimates of gray matter volume and tau pathology were standardized to facilitate the interpretation of the model output. We included age, sex, and years of education as covariates in all linear models; age and years of education were entered as centered, continuous numeric variables and sex was entered as a dummy‐coded numeric variable (female = 0, male = 1). Sensitivity analyses were also conducted to better understand the relationships between memory ability, metamemory, and anxiety and depression symptoms. In one set of sensitivity analyses we removed cognitive items from the GDS and recomputed the anxiety and depression composite score to determine if we still observed effects of anxiety and depression symptoms after accounting for this potential confound. In another set of analyses, we added a dummy‐coded covariate pertaining to psychiatric history (0 = no history, 1 = history of psychiatric illness); a small proportion of our sample had a history of psychiatric illness (7/72). In all the models described in this manuscript, the effects are reported as the estimate from the model with 95% confidence intervals and the corresponding test statistic and *p*‐value. For exploratory analyses, we report *p*‐values both with and without correction for multiple comparisons using false discovery rate correction. Model diagnostics, including a summary of variance inflation factors across all models, are described in the Supplement to ensure all models are robust to concerns of multicollinearity. The Supplement also contains Bayesian tests of the Probability of Direction to provide a complementary lens from which to view the results by quantifying the probability that the observed effect is positive or negative (i.e., an index similar to a frequentist *p*‐value). Data analysis was conducted in R version 4.4.2 (Team 2023) using the ‘easystats’ ecosystem (Lüdecke et al. 2019, 2020; Lüdecke, Ben‐Shachar, et al. 2021; Lüdecke, Patil, et al. 2021; Makowski et al. 2020).

## 3 Results

### 3.1 Metamemory Is Related to Anxiety and Depression Symptoms, but Not Memory Ability

**FIGURE 1:**
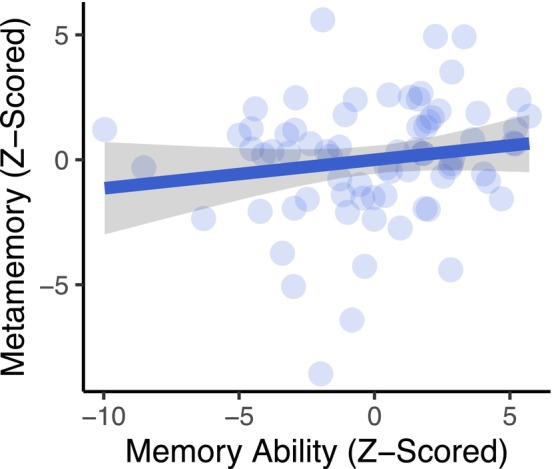
Model estimates (residualized) of metamemory as they relate to memory ability, controlling for age, sex, and years of education. Larger values represent better memory ability and higher levels of self‐reported metamemory. Error bands represent 95% confidence intervals. Data points represent individual participants.

**FIGURE 2:**
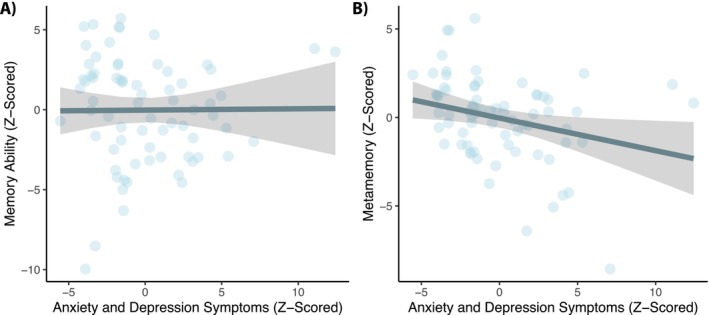
Anxiety and depression symptoms are not related to (A) memory ability, but are negatively associated with (B) metamemory. The plots include residualized model estimates controlling for age, sex, and years of education. In each plot, larger values represent higher levels of anxiety and depression symptoms and better memory ability and self‐reported metamemory. Error bands represent 95% confidence intervals. Data points represent individual participants.

We observed effects of age, but not sex or education, on memory ability (*B*_age_ = −0.96 [−1.71, −0.20], *t* = −2.53, *p* = 0.014; *B*_sex_ = −0.73 [−2.22, 0.76], *t* = −0.98, *p* = 0.332; *B*_edu_ = 0.59 [−0.16, 1.34], *t* = 1.57, *p* = 0.121), whereas we found that sex, but not age or education, was associated with metamemory (*B*_age_ = −0.46 [−1.02, 0.09], *t* = −1.66, *p* = 0.102; *B*_sex_ = 1.21 [0.11, 2.32], *t* = 2.19, *p* = 0.032; *B*_edu_ = 0.31 [−0.24, 0.87], *t* = 1.13, *p* = 0.262). These results suggest that higher age is associated with worse memory ability and that men tend to have higher metamemory scores than women despite a lack of observable sex differences in objective measures of memory ability. Nevertheless, and most critically, when we tested for the associations between objective and subjective measures of memory, we did not find reliable evidence that memory ability was related to metamemory, *B* = 0.11 [−0.06, 0.29], *t* = 1.29, *p* = 0.202, even when controlling for age, sex, and years of education (Figure 1). Next, we assessed the relationships between anxiety and depression symptoms and objective and subjective assessments of cognition to better understand the mechanisms that underlie these distinct constructs. Indeed, we found that anxiety and depressive symptoms were associated with metamemory, *B* = −0.19 [−0.35, −0.03], *t* = −2.38, *p* = 0.020 (Figure 2b), but not memory ability, *B* = 0.01 [−0.22, 0.23], *t* = 0.05, *p* = 0.957 (Figure 2a). More specifically, higher levels of anxiety and depression symptoms were associated with worse metamemory, but there was no association between memory ability and anxiety and depression symptoms. Neither removing cognitive‐related items from the anxiety and depression symptom composite score, nor controlling for individual differences in psychiatric history, changed the above reported effects, *ps* < 0.039.

To better understand which measures contained within the anxiety and depression symptom composite help to explain variance in metamemory, we entered each facet of the anxiety and depression composite as simultaneous predictors in a linear model of metamemory. Interestingly, we found evidence for the importance of depressive symptoms, as measured by the GDS, on metamemory. Higher GDS scores were associated with worse self‐reported metamemory scores, *B* = −0.88 [−1.72, −0.03], *t* = −2.07, *p* = 0.043, Further, the anxious arousal sub‐scale from the MASQ was also a significant predictor, *B* = −0.77 [−1.35, −0.18], *t* = −2.62, *p* = 0.011, which suggests that somatic sensations are also a component that contributes to the relationship between negative affect and metamemory. There were no other significant predictors observed in the model, *ps* > 0.093. Taken together, these findings suggest that affective dimensions, such as anxiety and depression symptoms, play a role in participants' perceptions of their own memory, but neither metamemory, nor anxiety and depression symptoms are reliably associated with objectively assessed memory ability.

### 3.2 Hippocampal Volume Is Associated With Memory Ability, but Not Metamemory

**FIGURE 3:**
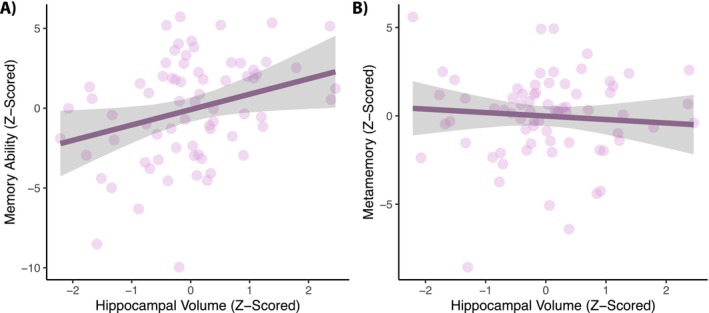
Estimates of hippocampal volume are associated (A) memory ability, but not (B) metamemory. In all plots, residualized model estimates are shown; larger values represent both greater gray matter volume and better memory ability and metamemory. Error bands represent 95% confidence intervals. Data points represent individual participants.

Given the lack of robust associations between memory ability and metamemory, we sought to examine the potential neural substrates that are associated with these functions. To accomplish this, we first examined the relationships between gray matter volume in the entorhinal cortex and hippocampus with both memory ability and metamemory. Entorhinal cortex volume was not associated with either memory ability, *B* = 0.42 [−0.40, 1.23], *t* = 1.02, *p* = 0.313, or metamemory, *B* = −0.36 [−0.97, 0.24], *t* = −1.20, *p* = 0.236. In contrast, hippocampal volume was associated with memory ability, *B* = 0.96 [0.10, 1.82], *t* = 2.23, *p* = 0.029 (Figure 3a), but not metamemory, *B* = −0.29[−0.95, 0.36], *t* = −0.89, *p* = 0.377 (Figure 3b). Namely, higher ICV‐adjusted estimates of hippocampal volume were associated with better memory ability, whereas no such relationship was observed between hippocampal volume and metamemory. The relationships between hippocampal volume and memory ability, *B* = 0.98 [0.10, 1.88], *t* = 2.23, *p* = 0.029, and metamemory, *B* = −0.19[−0.84, 0.45], *t* = −0.60, *p* = 0.553, remained after adding anxiety and depression symptoms as a covariate into the models. When viewed together, these findings suggest that dissociable mechanisms are associated with memory ability and metamemory. More specifically, memory ability, but not metamemory, is positively associated with hippocampal volume, whereas metamemory, but not memory ability, is more tightly coupled with anxiety and depression symptoms.

### 3.3 Associations Between Anxiety and Depression Symptoms and Metamemory Are Moderated by Hippocampal Tau Pathology

Given the relevance of both objective and subjective measures of cognition to AD, we also examined the extent to which tau burden in the entorhinal cortex and hippocampus was associated with memory ability and metamemory. Although both entorhinal, *r* = 0.31 [0.08, 0.50], *t* = 2.72, *p* = 0.008, and hippocampal tau burden, *r* = 0.26 [0.03, 0.406], *t* = 2.24, *p* = 0.028, were positively associated with age, we did not observe reliable associations between memory ability and tau burden in either the entorhinal cortex, *B* = −0.00 [−0.84, 0.83], *t* = −0.01, *p* = 0.992, or hippocampus, *B* = −0.43 [−1.21, 0.35], *t* = −1.11, *p* = 0.271, when controlling for age, sex, and education. Moreover, we also did not find evidence for direct associations between metamemory and tau burden in either the entorhinal cortex, *B* = −0.02 [−0.62, 0.59], *t* = −0.06, *p* = 0.953, or hippocampus, *B* = −0.22 [−0.78, 0.34], *t* = −0.79, *p* = 0.431; there also were no direct associations between anxiety and depression symptoms and entorhinal tau, *B* = −0.24 [−1.15, 0.67], *t* = −0.53, *p* = 0.597, or hippocampal tau, *B* = −0.31 [−1.17, 0.54], *t* = −0.74, *p* = 0.464, even when controlling for individual differences in psychiatric history or when removing cognitive items from the anxiety and depression symptom composite, *ps* > 0.322.

**FIGURE 4:**
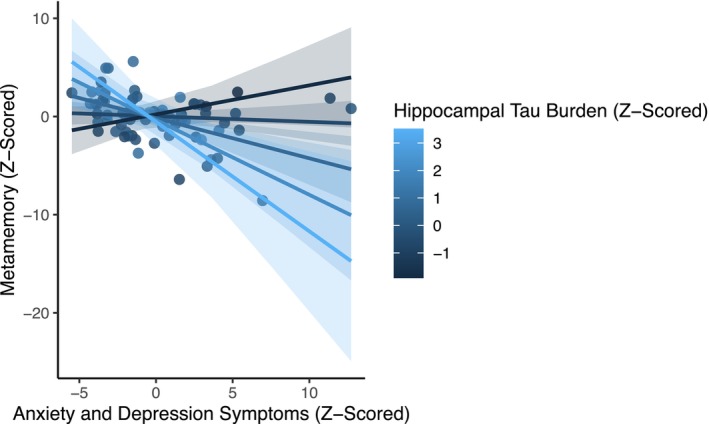
Associations between anxiety and depression symptoms and metamemory are moderated by hippocampal tau pathology. In each plot, residualized values are shown; larger numeric values represent greater levels of hippocampal tau pathology, greater number of anxiety and depression symptoms, and better metamemory. Error bands represent 95% confidence intervals. Data points represent individual participants.

Next, we sought to examine if tau pathology moderated the association between anxiety and depression symptoms and metamemory. In other words, because there is growing support for idea that depression and anxiety symptoms are related to tau pathology in the temporal lobe (Marchant et al. 2020; Markova et al. 2025), it is possible that the relationships between anxiety and depression symptoms and metamemory could be amplified in pathological aging. Indeed, we found that tau burden in the hippocampus moderated the association between anxiety and depression symptoms and metamemory, *B* = −0.26 [−0.46, −0.06], *t* = −2.61, *p* = 0.011 (Figure 4); this effect remained after controlling for individual differences in psychiatric history and after removing cognitive items from the anxiety and depression symptom composite, *ps* < 0.014. Specifically, individuals with high levels of both anxiety and depression symptoms and hippocampal tau burden had worse self‐reported metamemory. This effect was specific to hippocampal tau burden; the same pattern did not emerge for estimates of entorhinal tau burden and anxiety and depression symptoms, *B* = −0.01 [−0.22, 0.21], *t* = −0.05, *p* = 0.957. Interestingly, these analyses also revealed that when including both hippocampal tau burden and anxiety and depression symptoms, and their interaction, into the linear model of metamemory, there was a significant positive association between memory ability and metamemory, *B* = 0.022 [0.04, 0.40], *t* = 2.38, *p* = 0.020 (Table 2). This suggests that failing to account for measures of affect (e.g., anxiety and depression symptoms) and tau burden may obscure the positive association between objective and subjective memory measures in cognitively normal older adults. Collectively, these results suggest that how cognitively normal older adults think and feel about their memory is related not only to individual differences in anxiety and depression symptoms and underlying tau pathology, but also to objective assessments of memory ability, when controlling for affect and tau burden.

**TABLE 2:** Relationships between metamemory and memory ability, hippocampal tau burden, hippocampal volume, anxiety and depression symptoms when controlling for age, sex, and years of education.

Predictors	Metamemory			
	Estimates	CI	Statistic	*p*
Intercept	−0.56	−1.29 to 0.16	−1.56	0.125
Memory Ability	0.22	0.04 to 0.40	2.38	**0.020**
Anxiety and Depression Symptoms	−0.21	−0.37 to −0.05	−2.68	**0.009**
Hippocampal Tau	−0.15	−0.71 to 0.40	−0.55	0.581
Hippocampal Volume	−0.43	−1.07 to 0.22	−1.33	0.189
Age	−0.29	−0.94 to 0.36	−0.89	0.378
Sex	1.17	0.07 to 2.28	2.12	**0.038**
Education	0.21	−0.34 to 0.75	0.75	0.455
Anxiety and Depression Symptoms*Hippocampal Tau	−0.26	−0.46 to −0.06	−2.61	**0.011**
*R* ^2^/*R* ^2^ adjusted	0.289/0.197			

*Note:* Bolded *p*‐values represent significant effects.

### 3.4 Exploring the Contribution of Anxiety and Depression Symptoms and Tau Burden on Different Facets of Metamemory

As an extension of our findings detailed above, we sought to examine the extent to which the relationships between memory ability, metamemory, and anxiety and depression symptoms are consistent across each of the three dimensions of metamemory assessed in the current study. To accomplish this, we ran separate models using the contentment, ability, and strategy use sub‐scales of the MMQ. Replicating the finding that memory ability and metamemory are not reliably associated with each other, none of the metamemory sub‐scales were associated with memory ability, *ps* > 0.106 (Table 3). Interestingly, our findings suggest that observed associations between anxiety and depression symptoms and metamemory were likely driven by self‐reported memory contentment, *B* = −0.07 [−0.14, −0.01], *t* = −2.16, *p* = 0.035 (*p*_corrected_ = 0.194), and ability, *B* = −0.08 [−0.15, −0.02], *t* = −2.55, *p* = 0.013 (*p*_corrected_ = 0.194), but not strategy use, −0.03 [−0.10, 0.04], *t* = −0.96, *p* = 0.339 (*p*_corrected_ = 0.636). This pattern of results remained after controlling for individual differences in psychiatric history and when removing cognitive items in the anxiety and depression symptom composite, *ps* < 0.040. In other words, participants who had higher levels of anxiety and depression symptoms tended to have lower levels of contentment and worse perceptions of their memory ability, whereas anxiety and depression symptoms were unrelated to their usage of memory strategies in daily life contexts.

**TABLE 3:** Effects of memory ability on the three different facets of metamemory (contentment, ability, and strategy) when controlling for anxiety and depression symptoms, age, sex, and years of education.

Predictors	Metamemory: contentment				Metamemory: ability				Metamemory: strategy			
	Estimates	CI	Statistic	*p*	Estimates	CI	Statistic	*p*	Estimates	CI	Statistic	*p*
Intercept	−0.14	−0.45 to 0.16	−0.94	0.350	−0.08	−0.38 to 0.22	−0.52	0.604	−0.23	−0.55 to 0.08	−1.48	0.145
Memory Ability	0.06	−0.01 to 0.13	1.64	0.106	0.05	−0.02 to 0.13	1.55	0.125	0.00	−0.07 to 0.08	0.06	0.956
Anxiety and Depression Symptoms	−0.07	−0.14 to −0.01	−2.16	**0.035**	−0.08	−0.15 to −0.02	−2.55	**0.013**	−0.03	−0.10 to 0.04	−0.96	0.339
Age	0.01	−0.23 – 0.25	0.07	0.943	−0.13	−0.37 to 0.11	−1.09	0.281	−0.09	−0.34 to 0.16	−0.75	0.454
Sex	0.32	−0.14 to 0.78	1.37	0.174	0.17	−0.28 to 0.62	0.76	0.450	0.52	0.04 to 0.99	2.15	**0.035**
Education	0.10	−0.13 to 0.33	0.87	0.385	0.13	−0.09 to 0.36	1.17	0.247	−0.02	−0.26 to 0.22	−0.18	0.858
*R* ^2^/*R* ^2^ adjusted	0.156/0.093				0.197/0.137				0.101/0.034			

*Note:* Bolded *p*‐values represent significant effects.

Next, we probed the degree to which the moderating role of hippocampal tau on the relationship between anxiety and depression symptoms and metamemory was more closely related to distinct facets of metamemory. Consistent with the findings above, these sub‐scale analyses are consistent with a specific role of self‐reported memory contentment, *B* = −0.09 [−0.18, −0.01], *t* = −2.15, *p* = 0.036 (*p*_corrected_ = 0.122), and ability, *B* = −0.09 [−0.18, −0.01], *t* = −2.26, *p* = 0.027 (*p*_corrected_ = 0.137), but not strategy use measures, *B* = −0.08 [−0.16, 0.01], *t* = −1.74, *p* = 0.087 (*p*_corrected_ = 0.234) (Table 4). Taken together, these findings suggest that one's feelings of contentment about their memory and how they view their memory ability might be important facets of metamemory that have particular relevance to their anxiety and depression symptoms and underlying tau burden. Although these effects do not survive multiple comparisons correction, further exploring these facets of metamemory represents a prime target for future hypothesis testing.

**TABLE 4:** Associations between facets of metamemory (contentment, ability, and strategy) and memory ability, hippocampal tau burden, hippocampal volume, anxiety and depression symptoms when controlling for age, sex, and years of education.

Predictors	Metamemory: contentment				Metamemory: ability				Metamemory: strategy			
	Estimates	CI	Statistic	*p*	Estimates	CI	Statistic	*p*	Estimates	CI	Statistic	*p*
Intercept	−0.15	−0.46 to 0.16	−0.94	0.350	−0.09	−0.39 to 0.21	−0.60	0.548	−0.33	−0.64 to −0.01	−2.08	**0.042**
Memory Ability	0.09	0.02 to 0.17	2.39	**0.020**	0.09	0.02 to 0.17	2.47	**0.016**	0.03	−0.05 to 0.11	0.78	0.438
Anxiety and Depression Symptoms	−0.09	−0.16 to −0.02	−2.50	**0.015**	−0.09	−0.16 to −0.03	−2.83	**0.006**	−0.03	−0.10 to 0.03	−1.00	0.321
Hippocampal Tau	−0.06	−0.29 to 0.18	−0.46	0.645	0.05	−0.18 to 0.28	0.45	0.656	−0.15	−0.39 to 0.09	−1.24	0.218
Hippocampal Volume	−0.09	−0.36 to 0.19	−0.63	0.534	−0.06	−0.32 to 0.21	−0.43	0.671	−0.29	−0.56 to −0.01	−2.04	**0.046**
Age	−0.01	−0.29 to 0.27	−0.04	0.967	−0.15	−0.42 to 0.11	−1.15	0.255	−0.13	−0.41 to 0.15	−0.91	0.365
Sex	0.33	−0.14 to 0.81	1.40	0.168	0.21	−0.25 to 0.67	0.92	0.360	0.63	0.15 to 1.11	2.64	**0.011**
Education	0.08	−0.15 to 0.32	0.72	0.473	0.12	−0.11 to 0.34	1.04	0.301	0.00	−0.23 to 0.24	0.02	0.981
Anxiety and Depression Symptoms*Hippocampal Tau	−0.09	−0.18 to −0.01	−2.15	**0.036**	−0.09	−0.18 to −0.01	−2.26	**0.027**	−0.08	−0.16 to 0.01	−1.74	0.087
*R* ^2^/*R* ^2^ adjusted	0.228/0.129				0.283/0.190				0.204/0.102			

*Note:* Bolded *p*‐values represent significant effects.

## 4 Discussion

A clear takeaway from the present study is that affective variables are important to measure in conjunction with assessments of metamemory when studying aging and AD. Not only did anxiety and depression symptoms demonstrate strong linkages with metamemory, but our results further demonstrated that underlying hippocampal tau pathology exacerbated this negative relationship. Prior work has already shown that failing to account for anxiety and depression symptoms risks occluding the relationships between memory ability and metamemory (Burmester et al. 2016; Crumley et al. 2014). Our findings extend this further; we show that affective disturbances (i.e., anxiety and depression symptoms) are increasingly influential on metamemory in people harboring tau. This distinction is important because prior work has shown that the strength of the relationship between metamemory and negative affect is preserved across the adult life span (Rowell et al. 2016). In other words, by showing that tau pathology exacerbates the association between negative affect and metamemory, the data in the present study provide a clear demonstration that metamemory is worsened by affective disturbances specifically in pathological aging processes.

Critically, these results add to a growing body of work which has revealed that AD pathology is associated with behavioral phenotypes closely linked to neuropsychiatric symptoms, which include both anxiety and depression symptoms. Namely, analyses of postmortem tissue have shown that early stages of tau pathology in the entorhinal cortex and hippocampus (i.e., Braak I and II) are associated with increased prevalence of neuropsychiatric symptoms, including anxiety and depression symptoms (Ehrenberg et al. 2018). A similar pattern of results is reflected across studies that use in vivo imaging of tau pathology to examine its associations with both anxiety and depression symptoms, revealing consistent positive associations between tau pathology in temporal regions and greater levels of depression and anxiety symptoms (Gatchel et al. 2017; Marchant et al. 2020; Markova et al. 2025; Tissot et al. 2021). Directly relevant to the present study, recent work has also found that both anxiety and depression symptoms act synergistically with AD pathology in predicting cognitive decline across multiple domains, including memory ability, in cognitively unimpaired older adults (Pink et al. 2023). As such, gaining a better understanding of the ways in which tau pathology is associated with both affective variables and metacognition will be crucial for future work to tackle, as it may help to identify people earlier in the disease before cognition has begun to decline and direct them towards appropriate interventions.

Although it is possible that the lack of reliable associations between measures of memory ability and metamemory could be explained by the differences in assessment type—memory ability is often assessed with neuropsychological tests, whereas metamemory is commonly measured via retrospective self‐report measures—there is likely more to the story. Prior work has shown that when older adults are asked to make metacognitive judgments about their memory while performing memory ability assessments in the laboratory (i.e., metamemory assessments not reliant on retrospective recall that are tightly linked to the contents of the neuropsychological assessment), the two constructs diverge both behaviorally and with regard to their underlying neural mechanisms (Uquillas et al. 2020; Zhuang et al. 2022). This suggests that retrospective response biases from self‐report are likely not a strong contributor to the differences often observed between memory ability and metamemory. Moreover, in the current study, we did observe a positive association between memory ability, measured with neuropsychological assessments, and metamemory, indexed via retrospective self‐report, only after controlling for individual differences in anxiety and depression symptoms and hippocampal tau burden. This provides further evidence that the lack of strong associations between measures of memory ability and metamemory more likely stem from key differences in the constructs themselves, rather than the type of measurement used to assess them.

Indeed, the lack of consistent, positive associations between assessments of memory ability and metamemory observed in the literature could be better attributed to the different facets of memory function that these measures are intended to measure. Put another way, many of the retrospective self‐report measures of metamemory, including the MMQ used in the present study, were specifically designed to index daily life memory function (Hertzog et al. 1987; Troyer and Rich 2002). This distinction is important because many of the neuropsychological tasks commonly used to assess objective measures of memory ability in older adults fail to meaningfully translate to daily life (T. A. Salthouse 2004, 2012) or demonstrate sensitivity to preclinical AD pathology (Rentz et al. 2013). In contrast, repeated daily life assessments of memory ability, which offer the promise of both improved reliability and generalizability relative to one‐time laboratory assessments (Öhman et al. 2021; Polk et al. 2025; Sliwinski et al. 2018), do show greater sensitivity to AD biomarkers (Allard et al. 2014). By extension, it is possible that repeated daily life assessments of memory ability could yield stronger and more reliable associations with metamemory in older adults and should be a priority for future directions in this research area.

Nevertheless, when considering the complex relationships between memory ability and metamemory in aging, there are several limitations that could not be directly addressed in the current study. For one, the relationships between cognitive and metacognitive processes were only assessed in the domain of memory. Although prior work has shown that self‐reported metamemory is associated with metacognition in other domains (Payne et al. 2017), it will be crucial for future studies to disentangle both the behavioral and neural mechanisms underlying these relationships. Multi‐domain investigations will be particularly important as they relate to AD pathology, given the recent work suggesting that there are dissociations between beta‐amyloid and tau pathology on executive function versus memory ability (Chen et al. 2025; Pezzoli et al. 2025). Further, anxiety and depression symptoms were assessed in a healthy older adult sample, with broad self‐report assessments. As such, we cannot draw conclusions about how the relationships between affective and subjective cognitive variables are affected by clinical diagnoses, such as depression or anxiety, or also how other dimensions of health and well‐being (e.g., physical health, sleep) play a role in these relationships. Work that can expand the present work to understand the contributions of multiple health‐related metrics will be critical. Moreover, our sensitivity to detect effects as they pertain to the relationships between subjective and objective memory measures could be improved with longitudinal assessments, in contrast to the cross‐sectional assessments used in the present study. Indeed, prior work has found that self‐reported memory is related to participants' change in objectively assessed memory ability across different timepoints, rather than the memory score assessed only at the time of administration of the self‐reported memory questionnaire (Chen et al. 2019). The present work is also limited both in its sample size and in its diversity across race, ethnicity, and levels of education. Future work should aim to assess a larger and more diverse pool of participants on multi‐domain cognitive and affective measures across time to better elucidate the linkages between these constructs and to more rigorously test for the generalizability of these effects.

Despite its limitations, the current study helps to contribute to a growing literature on the relationships between memory ability and metamemory in aging. Namely, we used psychometrically validated behavioral measures and multimodal imaging to show that both shared and distinct mechanisms underlie memory ability and metamemory processes. In particular, the results suggest that assessments of affect are important to measure when assessing the relationships between memory ability and metamemory, especially in the context of AD pathology, providing a strong foundation for future investigations.

## Funding

This manuscript is the result of funding by the National Institutes of Health (NIH): R01‐AG074330 to A.S.B.; T32‐NS007292 and F32‐AG085890 to J.L.C. As such, it is subject to the NIH Public Access Policy. Through acceptance of this federal funding, NIH has been given a right to make this manuscript publicly available in PubMed Central upon the Official Date of Publication, as defined by NIH.

## Conflicts of Interest

J.M.H. is co‐founder of and equity holder in Eikonizo Therapeutics and Sensorium Therapeutics, where he also serves as CEO. He is currently an advisor to Rocket Science Health, Human Health, Delix Therapeutics, and Psy Therapeutics. All other authors report no competing interests.

## Supporting information


**Data S1:** Supporting Information.

## Data Availability

All data and analysis code are publicly available on the Open Science Framework: https://osf.io/6et32. Supporting Information provide additional descriptions and auxiliary analyses.
