# Supplementary material for: Dissociable Mechanisms Underlie Differences Between Memory and Metamemory in Older Adults: The Differentiating Role of Anxiety and Depression Symptoms
Source: Hippocampus. 2026 Apr 19;36(3):e70100. doi: 10.1002/hipo.70100 (PMC13092649; doi:10.1002/hipo.70100)
Supplement: Supplementary file 1 — Data S1: Supporting Information. [file HIPO-36-0-s001.docx]

Supplement: Dissociable mechanisms underlie differences between memory and metamemory in older adults: the differentiating role of anxiety and depression symptoms

J.L. Crawford et al., 2026-03-25

# Memory composite score

## Contains free recall measures from both short and long delays from the CVLT and visual reproduction tasks

Higher scores represent better memory ability. Variable naming conventions: CVLT_SD FR = short delay free recall, CVLT_LD FR = long delay free recall

*#merging data frames and cleaning data*

d.CVLT <- BABS.NP.sess1 %>% select(BABS_ID, starts_with("CVLT")) %>%

distinct(BABS_ID, .keep_all = T) %>% mutate_at(vars(starts_with("CVLT")), as.numeric) %>%

mutate(Learning_sum = CVLT_listA Trial1 + CVLT_listA Trial2 + CVLT_listA Trial3 + CVLT_listA Trial4 + CVLT_listA Trial5) %>%

select(-starts_with("CVLT_listA"))

d.CVLT.trim <- d.CVLT %>% filter(BABS_ID %**in**% sub.IDs) %>% mutate(Subject = parse_number(BABS_ID)) %>% sele ct(-BABS_ID) %>% select(Subject, Learning_sum, CVLT_listB, starts_with("CVLT_SD"), starts_with("CVLT_LD")) *#CVLT Summary*

CVLT.desc.sum <- d.CVLT.trim %>% describe() %>% select(-vars) %>% rownames_to_column(var = "Variable") %>% filter(Variable == "CVLT_SD FR" | Variable == "CVLT_LD FR") %>% select(-n)

kable(CVLT.desc.sum, col.names = c("Variable" "Mean" "SD" "Median" "Trimmed Mean" "Median Abs. Deviatio n" "Min" "Max" "Range" "Skew" "Kurtosis" "SE"),

caption = "CVLT Summary Table") %>% kable_styling()

CVLT Summary Table

| **Variable** | **Mean** | **SD** | **Median** | **Trimmed**  **Mean** | **Median**  **Abs. Deviation** | **Min** | **Max** | **Range** | **Skew** | **Kurtosis** | **SE** |
| --- | --- | --- | --- | --- | --- | --- | --- | --- | --- | --- | --- |
| CVLT_SD FR | 11.39726 | 3.090037 | 12 | 11.57627 | 2.9652 | 3 | 16 | 13 | -0.5250626 | -0.3033732 | 0.3616614 |
| CVLT_LD FR | 12.01370 | 3.052745 | 12 | 12.28814 | 2.9652 | 4 | 16 | 12 | -0.6541676 | -0.1228930 | 0.3572968 |

Variable naming conventions: WMS_VR1 = short delay free recall, WMS_VR2 = long delay free recall

d.VR.trim <- BABS.NP.sess1 %>% filter(BABS_ID %**in**% sub.IDs) %>% mutate(Subject = parse_number(BABS_ID)) %>% select(-BABS_ID) %>% select(Subject, starts_with("WMS_V"))

*#WMS Summary*

WMS.desc.sum <- d.VR.trim %>% describe() %>% select(-vars) %>% rownames_to_column(var = "Variable") %>% filter(Variable == "WMS_VR1" | Variable == "WMS_VR2") %>% select(-n)

kable(WMS.desc.sum, col.names = c("Variable" "Mean" "SD" "Median" "Trimmed Mean" "Median Abs. Deviation" "M in" "Max" "Range" "Skew" "Kurtosis" "SE"),

caption = "Visual Reproduction Summary Table") %>% kable_styling()

| Visual Reproduction Summary Table |  |  |  |  | | | | | |
| --- | --- | --- | --- | --- | --- | --- | --- | --- | --- |
|  |  | **Trimmed** | **Median**  **Abs.** |  |  |  |  |  |  |
| **Variable Mean SD** | **Median** | **Mean** | **Deviation** | **Min** | **Max** | **Range** | **Skew** | **Kurtosis** | **SE** |
| WMS_VR1 75.15068 15.06253 | 75 | 75.81356 | 16.3086 | 34 | 103 | 69 | -0.3374116 | -0.5243164 | 1.762936 |
| WMS_VR2 55.90411 21.93625 | 55 | 55.84746 | 25.2042 | 9 | 97 | 88 | 0.0912729 | -0.8764513 | 2.567444 |


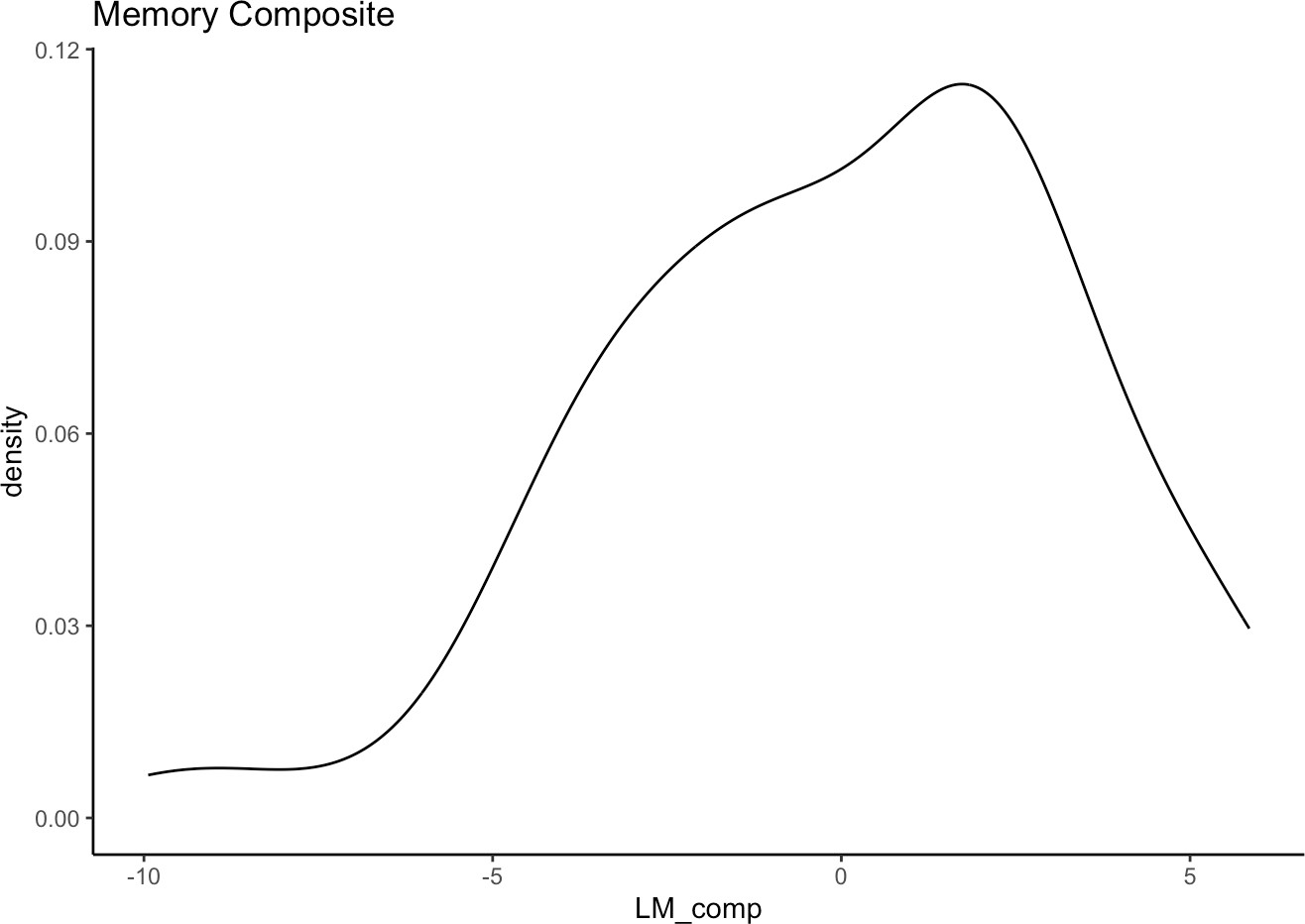


*#creating data frame with all WM measures*

d.LM <- d.CVLT.trim %>% inner_join(d.VR.trim) %>% drop_na()

d.LM.comp <- d.LM %>% mutate(across(Learning_sum:WMS_VR2, ~ c(scale(.))),

LM_comp = CVLT_SD FR + CVLT_LD FR + WMS_VR1 + WMS_VR2) %>% select(Subject, L

M_comp)

d.cog.comp <- d.LM.comp

*#plot distribution of composite*

p.LM.comp <- ggplot(d.LM.comp, aes(LM_comp)) + geom_density() +

labs(title = "Memory Composite") + theme_classic()

p.LM.comp

*#plot correlational structure of items in the memory composite*

corr.LM.comp <- d.LM %>% mutate(across(Learning_sum:WMS_VR2, ~ c(scale(.)))) %>% select(CVLT_SD FR, CVLT_LD FR, WMS_VR1, WMS_VR2) %>% correlation()

corr.LM.comp %>% summary(redundant = F) %>%

plot() + ggplot2::theme(axis.text.x = element_text(angle = 45, vjust = 1, hjust=1))


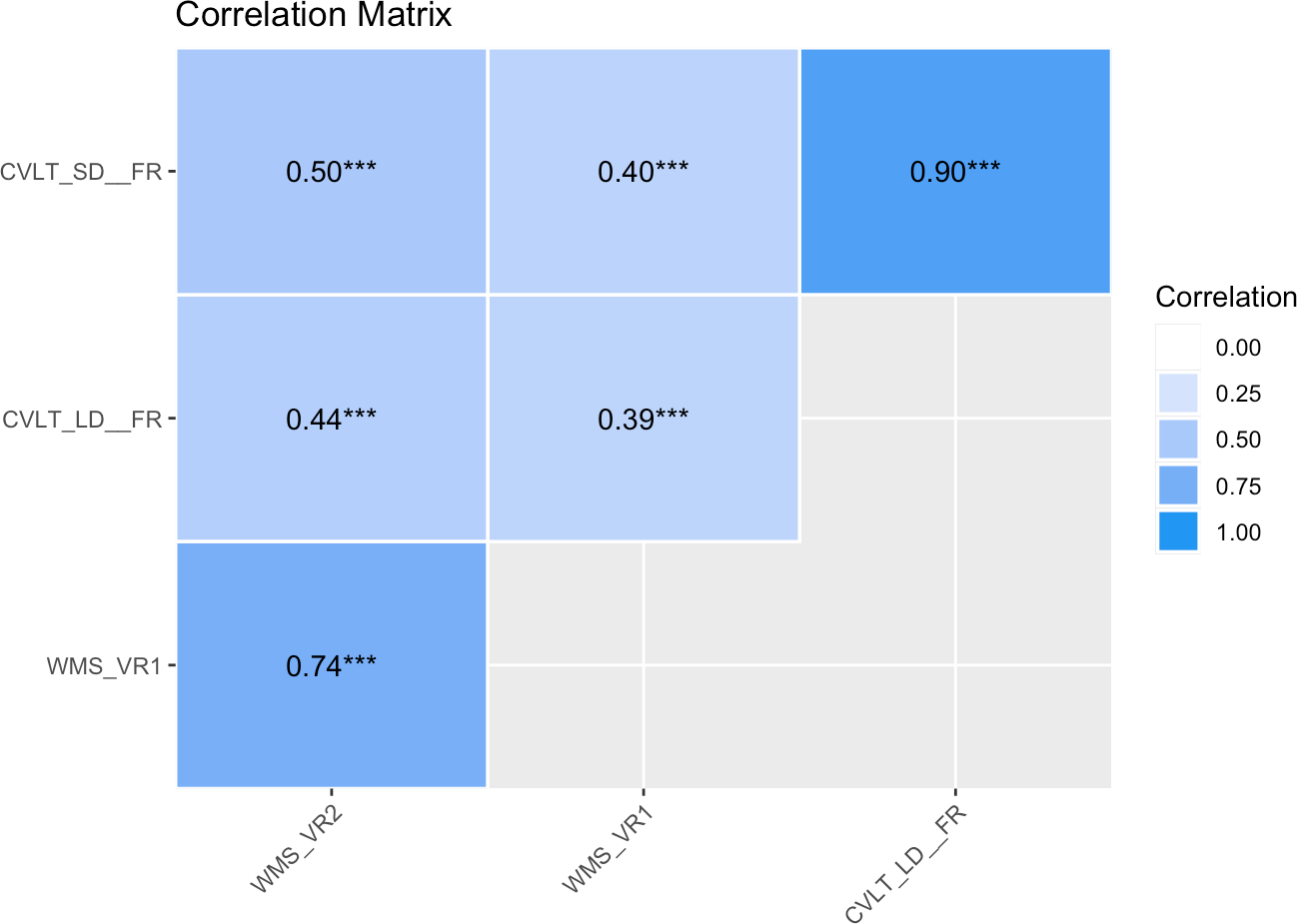


# Metamemory composite score

## Includes the three sub-scales from the MMQ: general contentment, ability, and strategy

Higher scores indicate better self-reported metamemory function. Variable naming conventions: Gen = general memory contentment, Able

= memory ability in daily life contexts, Strat = memory strategy use in daily life contexts

*#import metamemory and examine correlation across domains*

d.metamem.trim <- Metamem %>% filter(BABS_ID %**in**% sub.IDs & Metamemory_Session == 1) %>% mutate(Subject = p arse_number(BABS_ID)) %>% select(-BABS_ID) %>% select(-Metamemory_Session)

*#Metamemory Summary*

metamem.desc.sum <- d.metamem.trim %>% describe() %>% select(-vars) %>% rownames_to_column(var = "Variabl e") %>%

filter(Variable != "Subject") %>% select(-n)

kable(metamem.desc.sum, col.names = c("Variable" "Mean" "SD" "Median" "Trimmed Mean" "Median Abs. Deviatio n" "Min" "Max" "Range" "Skew" "Kurtosis" "SE"),

caption = "Metamemory Summary Table") %>% kable_styling()

| Metamemory Summary | Table |  |  |  |  | | | | | |
| --- | --- | --- | --- | --- | --- | --- | --- | --- | --- | --- |
|  |  |  | **Trimmed** | **Median**  **Abs.** |  |  |  |  |  |  |
| **Variable Mean** | **SD** | **Median** | **Mean** | **Deviation** | **Min** | **Max** | **Range** | **Skew** | **Kurtosis** | **SE** |
| Able 78.09722 | 9.963539 | 78.5 | 78.53448 | 9.6369 | 46 | 99 | 53 | -0.4773247 | 0.5224550 | 1.174214 |
| Gen 73.27778 | 11.427940 | 76.5 | 74.62069 | 11.1195 | 34 | 89 | 55 | -1.0149750 | 0.7392698 | 1.346796 |
| Strat 65.05556 | 9.226247 | 65.5 | 64.96552 | 9.6369 | 47 | 87 | 40 | 0.0412524 | -0.4320397 | 1.087324 |

*#plotting correlational structure of metamemory questionnaire* corr.metamem <- d.metamem.trim %>% select(-Subject) %>% correlation() corr.metamem %>%

summary(redundant = F) %>%

plot() + ggplot2::theme(axis.text.x = element_text(angle = 45, vjust = 1, hjust=1))


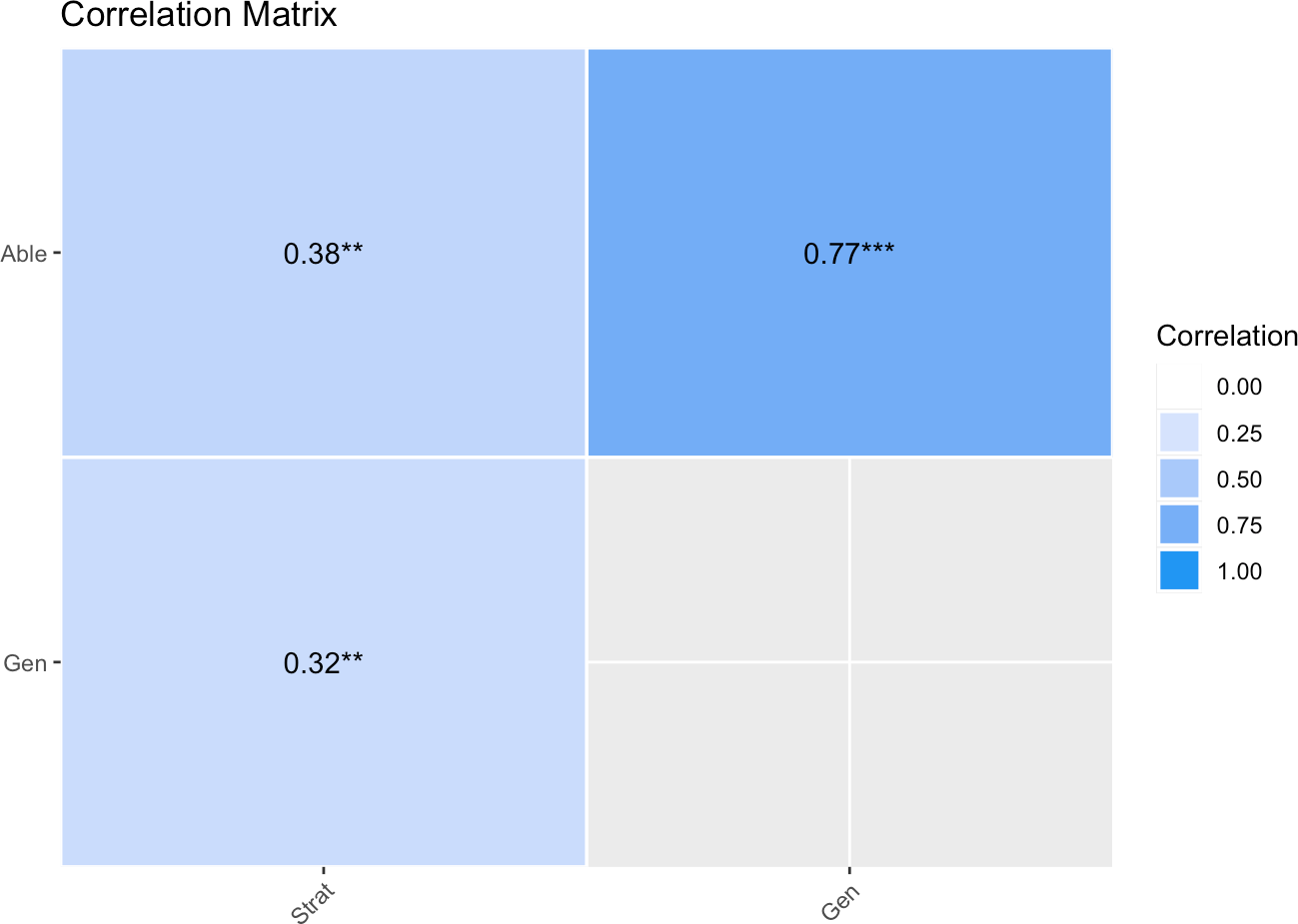


*#Metamemory*

metamem.comp <- d.metamem.trim %>% mutate(across(Able:Strat, ~ c(scale(.))),

metamem_comp = Able + Gen + Strat) d.metacog.comp <- metamem.comp

d.cog.meta <- d.cog.comp %>% inner_join(d.metacog.comp) %>% inner_join(BABS.demo)

*#plot distribution of composite*

p.metamem.comp <- ggplot(d.metacog.comp, aes(metamem_comp)) + geom_density() +

labs(title = "Metamemory Composite") + theme_classic()

p.metamem.comp


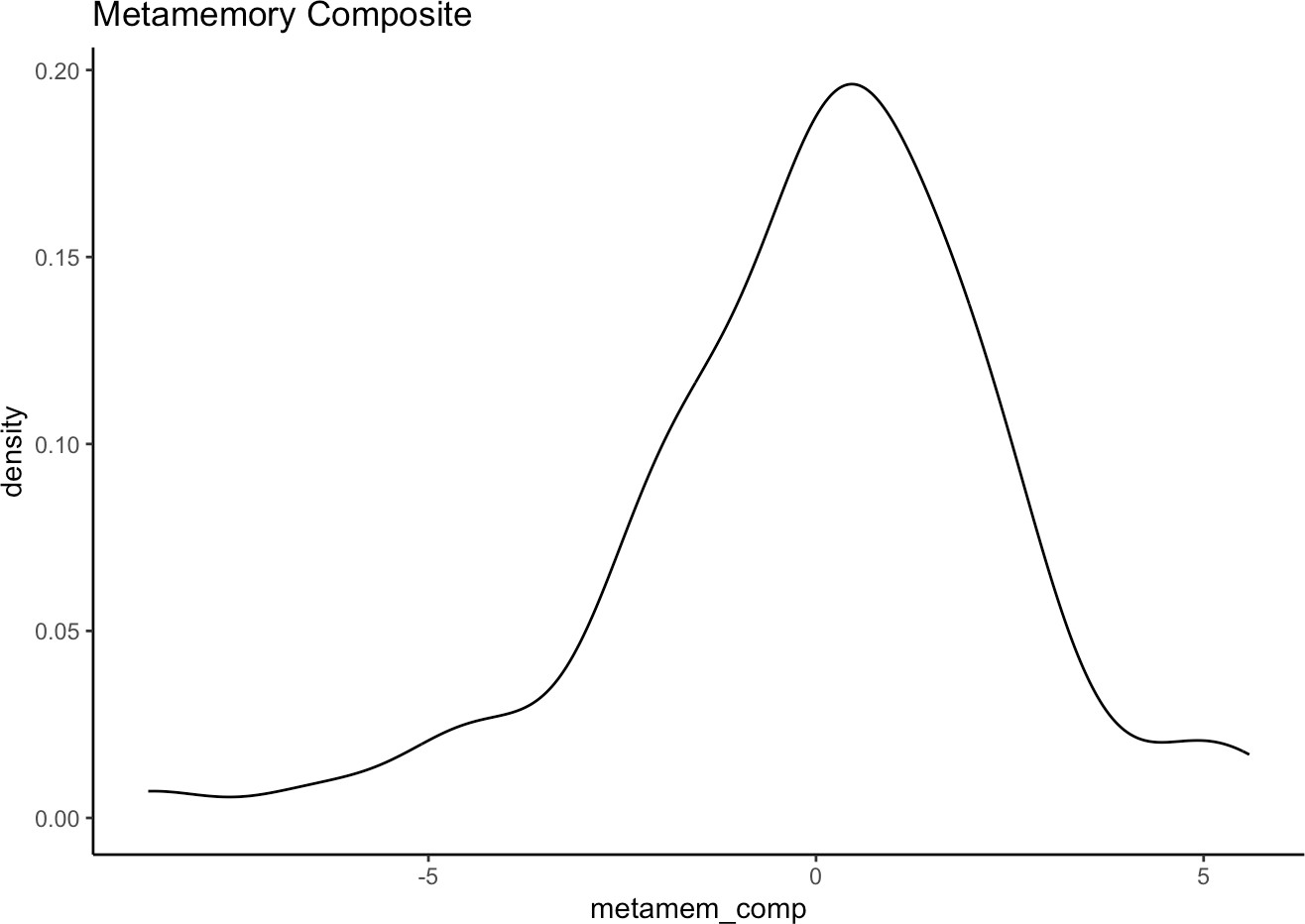


# Anxiety and depression symptoms composite score

## Depression and anxiety symptom questionnaires: GDS, MASQ, PSWQ

Larger scores represent a greater number of depression or anxiety symptoms. Variable naming conventions: GDS = GDS total score, AA = anxious arousal sub-scale of MASQ, GD = general distress sub-scale of MASQ, AD = anhedonic depression sub-scale of MASQ, PSWQ = PSWQ total score

*#import GDS*

d.GDS.trim <- GDS %>% filter(BABS_ID %**in**% sub.IDs) %>% mutate(Subject = parse_number(BABS_ID)) %>% select(-BABS_ID) %>% select(Subject, GDS) %>% mutate(GDS = as.numeric(GDS))

*#GDS Summary*

GDS.desc.sum <- d.GDS.trim %>% describe() %>% select(-vars) %>% rownames_to_column(var = "Variable") %>% filter(Variable != "Subject") %>% select(-n)

kable(GDS.desc.sum, col.names = c("Variable" "Mean" "SD" "Median" "Trimmed Mean" "Median Abs. Deviation" "M in" "Max" "Range" "Skew" "Kurtosis" "SE"),

caption = "GDS Summary Table") %>% kable_styling()

GDS Summary Table

**Variable Mean SD Median**

**Trimmed**

**Mean**

**Median Abs.**

**Deviation Min Max Range Skew Kurtosis SE**

GDS 2.808219 3.195733 2 2.220339 2.9652 0 14 14 1.574632 2.13295 0.3740323

*#import MASQ and examine correlation across domains*

d.MASQ.trim <- MASQ %>% filter(BABS_ID %**in**% sub.IDs & MASQ_Session == 1) %>% mutate(Subject = parse_number (BABS_ID)) %>% select(-BABS_ID) %>% select(-MASQ_Session)

*#MASSQ Summary*

MASQ.desc.sum <- d.MASQ.trim %>% describe() %>% select(-vars) %>% rownames_to_column(var = "Variable") %>% filter(Variable != "Subject") %>% select(-n)

kable(MASQ.desc.sum, col.names = c("Variable" "Mean" "SD" "Median" "Trimmed Mean" "Median Abs. Deviatio n" "Min" "Max" "Range" "Skew" "Kurtosis" "SE"),

caption = "MASQ Summary Table") %>% kable_styling()

| MASQ Summary Table |  | | | | | | | | | |
| --- | --- | --- | --- | --- | --- | --- | --- | --- | --- | --- |
| **Variable Mean** | **SD** | **Median** | **Trimmed**  **Mean** | **Median Abs. Deviation** | **Min** | **Max** | **Range** | **Skew** | **Kurtosis** | **SE** |
| AA 11.80822 | 1.883094 | 11 | 11.54237 | 1.4826 | 10 | 17 | 7 | 0.9875861 | 0.027301 | 0.2203995 |
| AD 25.19178 | 6.800036 | 25 | 25.08475 | 7.4130 | 10 | 45 | 35 | 0.2258435 | -0.0424222 | 0.7958840 |
| GD 14.09589 | 4.038579 | 13 | 13.45763 | 2.9652 | 10 | 30 | 20 | 1.7966380 | 3.9687075 | 0.4726799 |

*#MASQ*

MASQ.comp <- d.MASQ.trim %>% mutate(across(AA:GD, ~ c(scale(.))),

MASQ_comp = AA + AD + GD) %>% select(Subject, MASQ_comp)

*#import PSWQ*

d.PSWQ.trim <- PSWQ %>% filter(BABS_ID %**in**% sub.IDs & PSWQ_Session == 1) %>% mutate(Subject = parse_number (BABS_ID)) %>% select(-BABS_ID) %>% select(-PSWQ_Session)

*#PSWQ Summary*

PSWQ.desc.sum <- d.PSWQ.trim %>% describe() %>% select(-vars) %>% rownames_to_column(var = "Variable") %>% filter(Variable != "Subject") %>% select(-n)

kable(PSWQ.desc.sum, col.names = c("Variable" "Mean" "SD" "Median" "Trimmed Mean" "Median Abs. Deviatio n" "Min" "Max" "Range" "Skew" "Kurtosis" "SE"),

caption = "PSWQ Summary Table") %>% kable_styling()

PSWQ Summary Table

**Variable Mean SD Median**

**Trimmed**

**Mean**

**Median Abs.**

**Deviation Min Max Range Skew Kurtosis SE**

PSWQ 35.26761 12.28932 35 34.14035 13.3434 17 74 57 0.8163472 0.3967653 1.458474

*#combine dataframes and examine correlation across questionnaires*

d.anxdep <- d.GDS.trim %>% inner_join(d.MASQ.trim) %>% inner_join(d.PSWQ.trim) *#plotting correlational structure of anxiety and depression measures* corr.anxdep <- d.anxdep %>% select(-Subject) %>% correlation()

corr.anxdep %>% summary(redundant = F) %>%

plot() + ggplot2::theme(axis.text.x = element_text(angle = 45, vjust = 1, hjust=1))


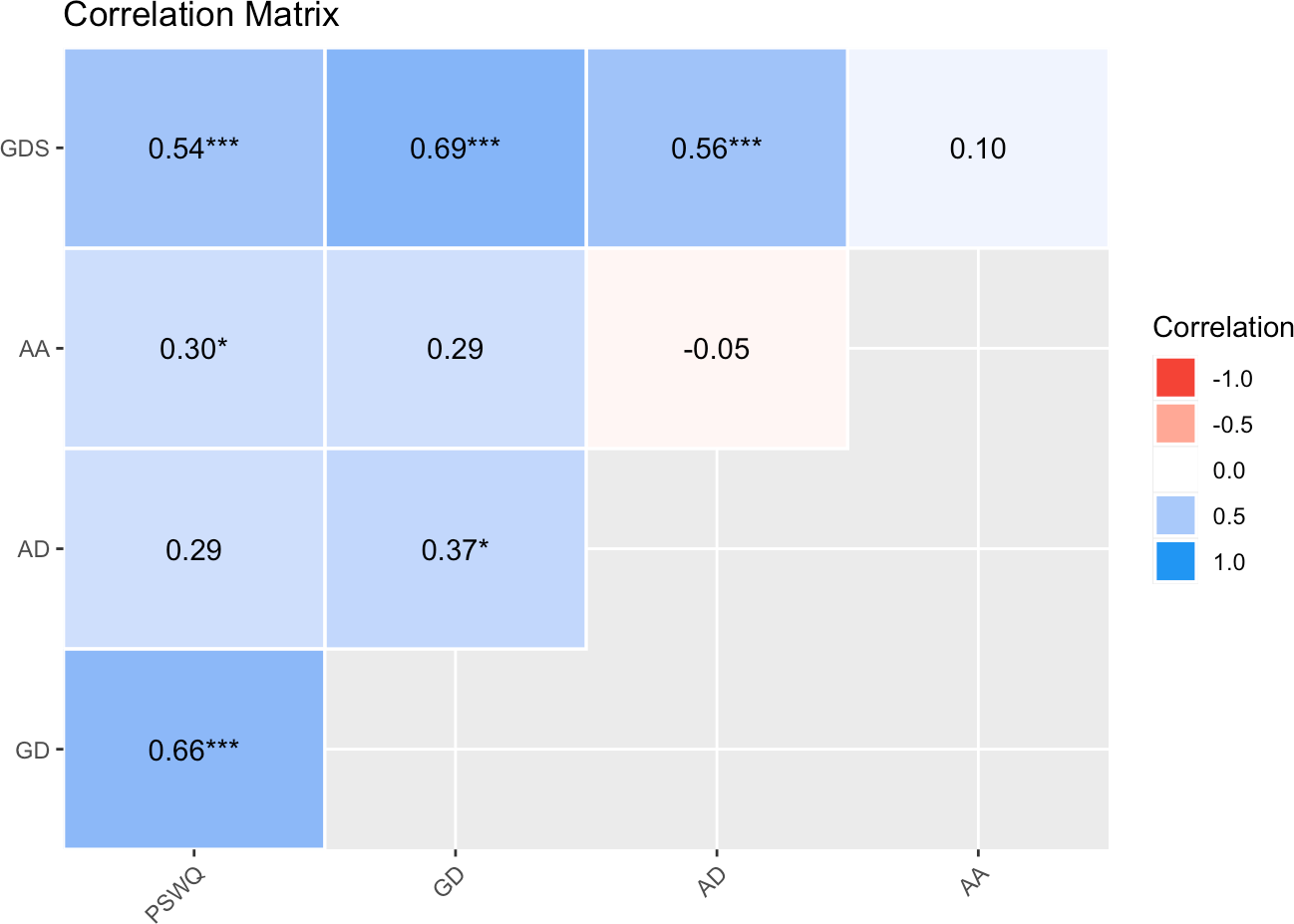


*#create composite*

anxdep.comp <- d.anxdep %>% mutate(across(GDS:PSWQ, ~ c(scale(.))),

anxdep_comp = GDS + AA + AD + GD + PSWQ) %>% select(Subject, anxdep_comp, GDS, AA, AD, GD, PSWQ) %>% drop_na()

d.cog.meta.anx <- d.cog.meta %>% inner_join(anxdep.comp)

*#plot distribution of composite*

p.anxdep.comp <- ggplot(d.cog.meta.anx, aes(anxdep_comp)) + geom_density() +

labs(title = "Anxiety and Depression Symptom Composite") + theme_classic()

p.anxdep.comp


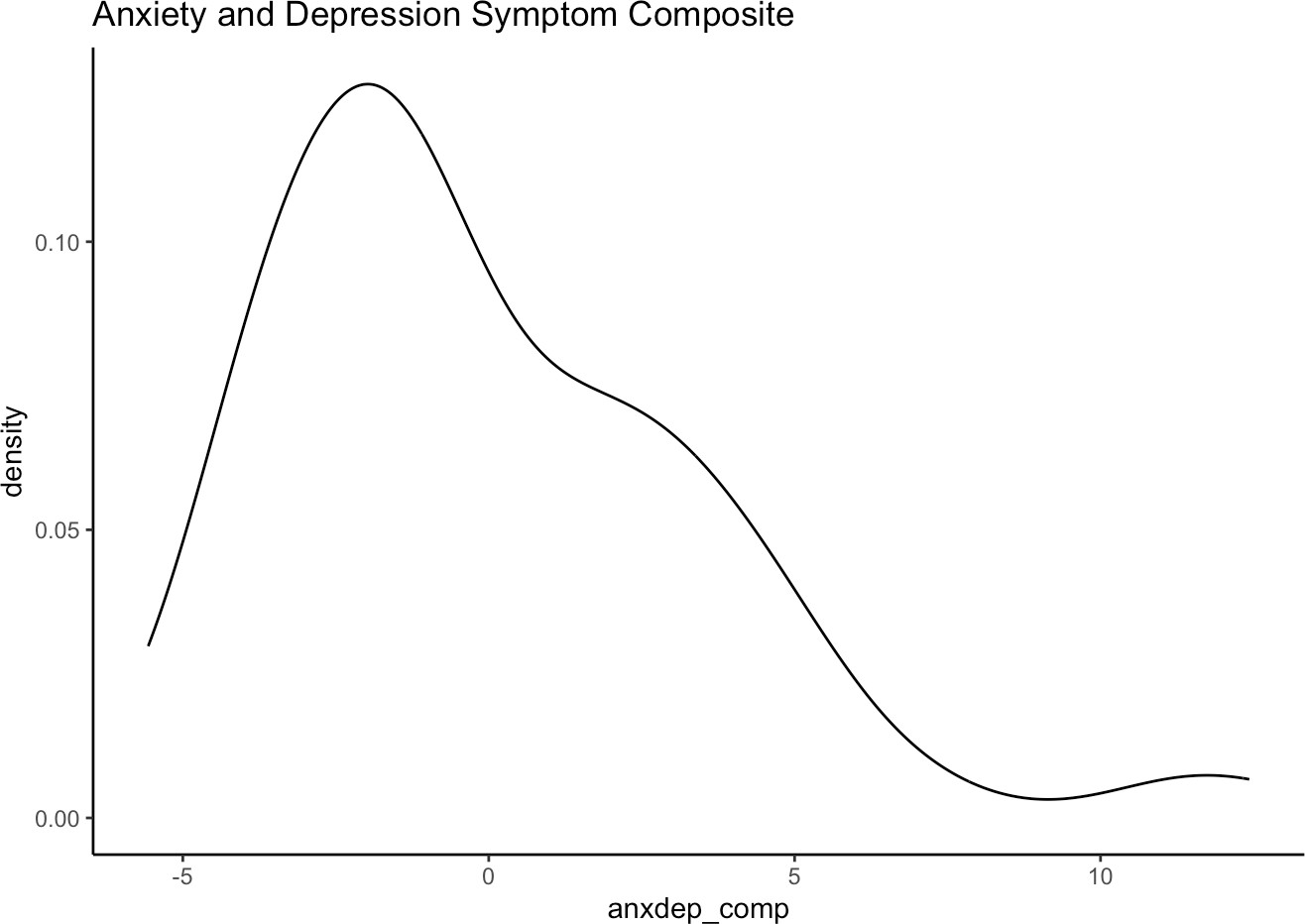


# Summarizing model diagnostics from behavioral analyses


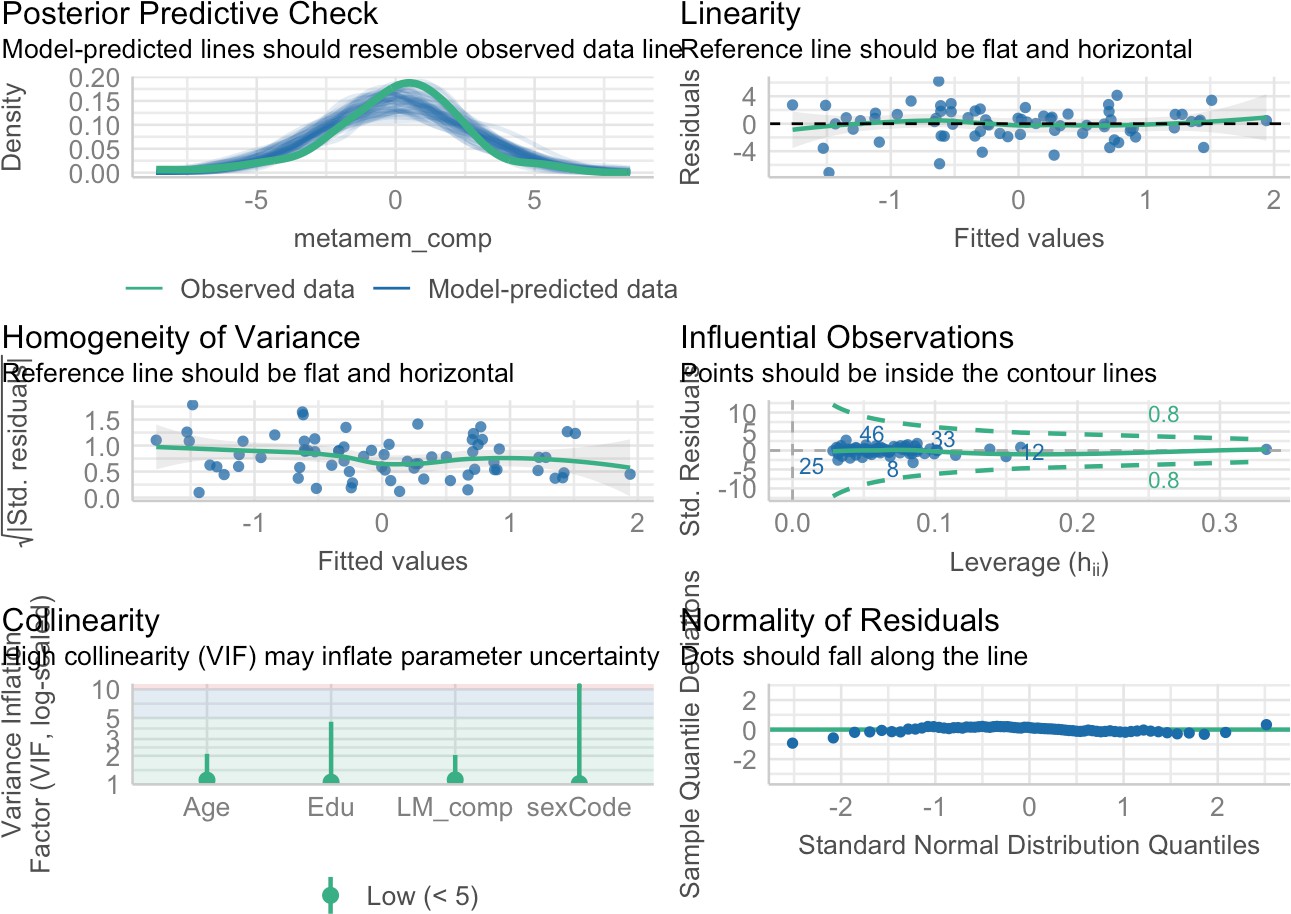


*#relationship between cognitive function and self-reported memory*

m.metamem.cog <- lm(data = d.cog.meta, metamem_comp ~ LM_comp + Age + sexCode + Edu) check_model(m.metamem.cog)

bootstrap_parameters(m.metamem.cog, test = "pd")

## # Fixed Effects

##

## Parameter

##

| Coefficient |

95% CI |

pd

## (Intercept) | 3.15 | [-2.88, 9.64] | 83.90%

## LM_comp | 0.12 | [-0.02, 0.28] | 96.30%

## Age | -0.07 | [-0.16, 0.02] | 93.80%

## sexCode | 1.22 | [ 0.16, 2.30] | 99.30%

## Edu | 0.06 | [-0.09, 0.23] | 77.80%


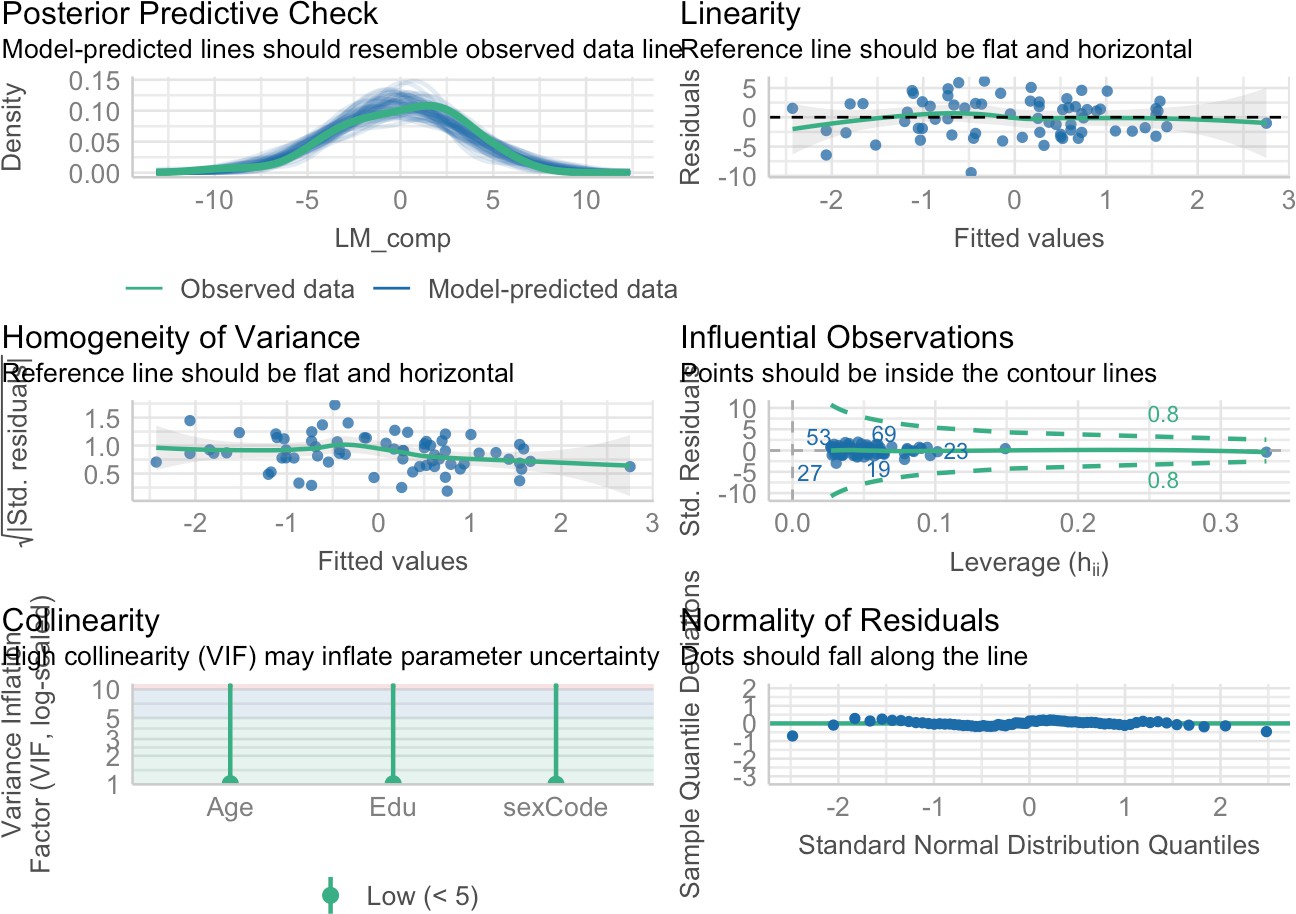


*#basic descriptive models*

m.LM.covariates <- lm(data = d.cog.meta.anx, LM_comp ~ Age + sexCode + Edu) check_model(m.LM.covariates)

bootstrap_parameters(m.LM.covariates, test = "pd")

## # Fixed Effects

##

## Parameter

##

| Coefficient |

95% CI |

pd

## (Intercept) | 8.85 | [ 1.52, 17.29] | 98.60%

## Age | -0.16 | [-0.28, -0.05] | 99.70%

## sexCode | -0.66 | [-2.16, 0.79] | 79.10%

## Edu | 0.14 | [-0.02, 0.33] | 95.90%

m.metamem.covariates <- lm(data = d.cog.meta.anx, metamem_comp ~ Age + sexCode + Edu) check_model(m.metamem.covariates)


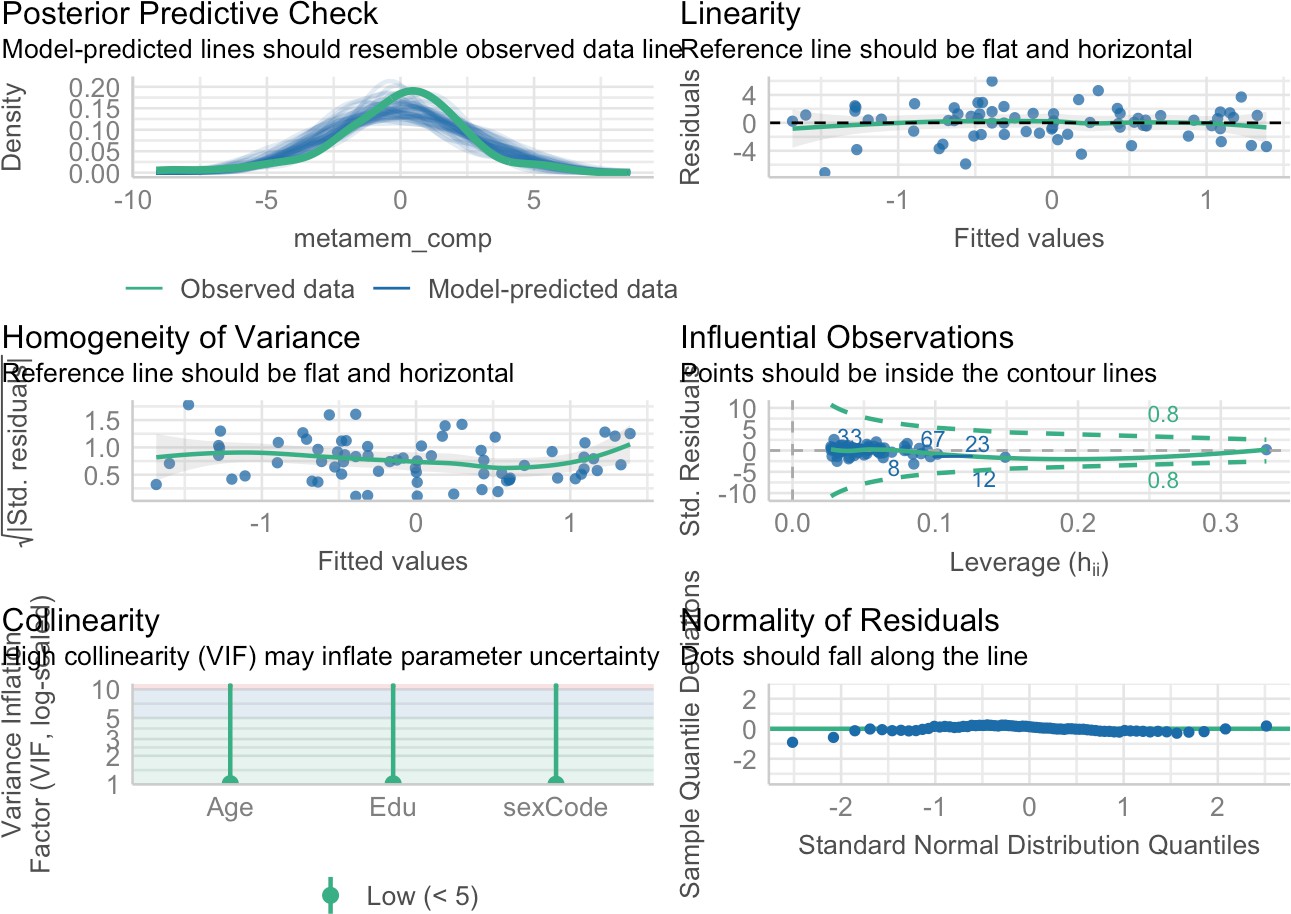


bootstrap_parameters(m.metamem.covariates, test = "pd")

## # Fixed Effects

##

## Parameter

##

| Coefficient |

95% CI |

pd

## (Intercept) | 3.26 | [-2.75, 9.80] | 84.10%

## Age | -0.08 | [-0.17, 0.01] | 95.90%

## sexCode | 1.17 | [ 0.14, 2.28] | 98.30%

## Edu | 0.08 | [-0.07, 0.25] | 85.90%

m.anxdep.covariates <- lm(data = d.cog.meta.anx, anxdep_comp ~ Age + sexCode + Edu) check_model(m.anxdep.covariates)


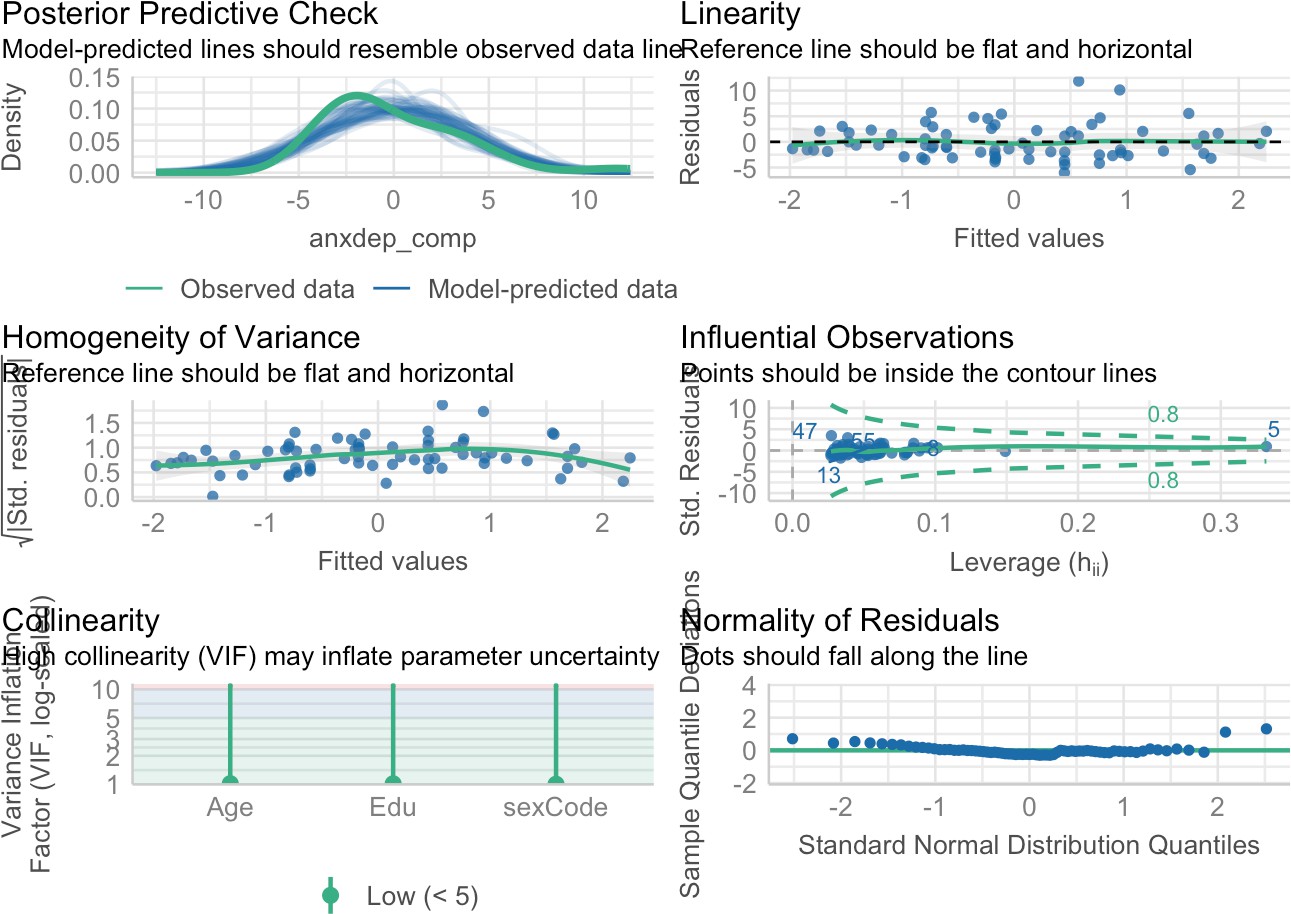


bootstrap_parameters(m.anxdep.covariates, test = "pd")

## # Fixed Effects

##

## Parameter

##

| Coefficient |

95% CI |

pd

## (Intercept) | -6.65 | [-15.97, 3.36] | 92.10%

## Age | 0.12 | [ -0.02, 0.24] | 96.80%

## sexCode | -1.66 | [ -3.12, -0.08] | 97.90%

## Edu | -0.06 | [ -0.28, 0.10] | 74.80%

*#relationship between anxiety and depression and memory ability*

m.LM.anx <- lm(data = d.cog.meta.anx, LM_comp ~ anxdep_comp + Age + sexCode + Edu) check_model(m.LM.anx)


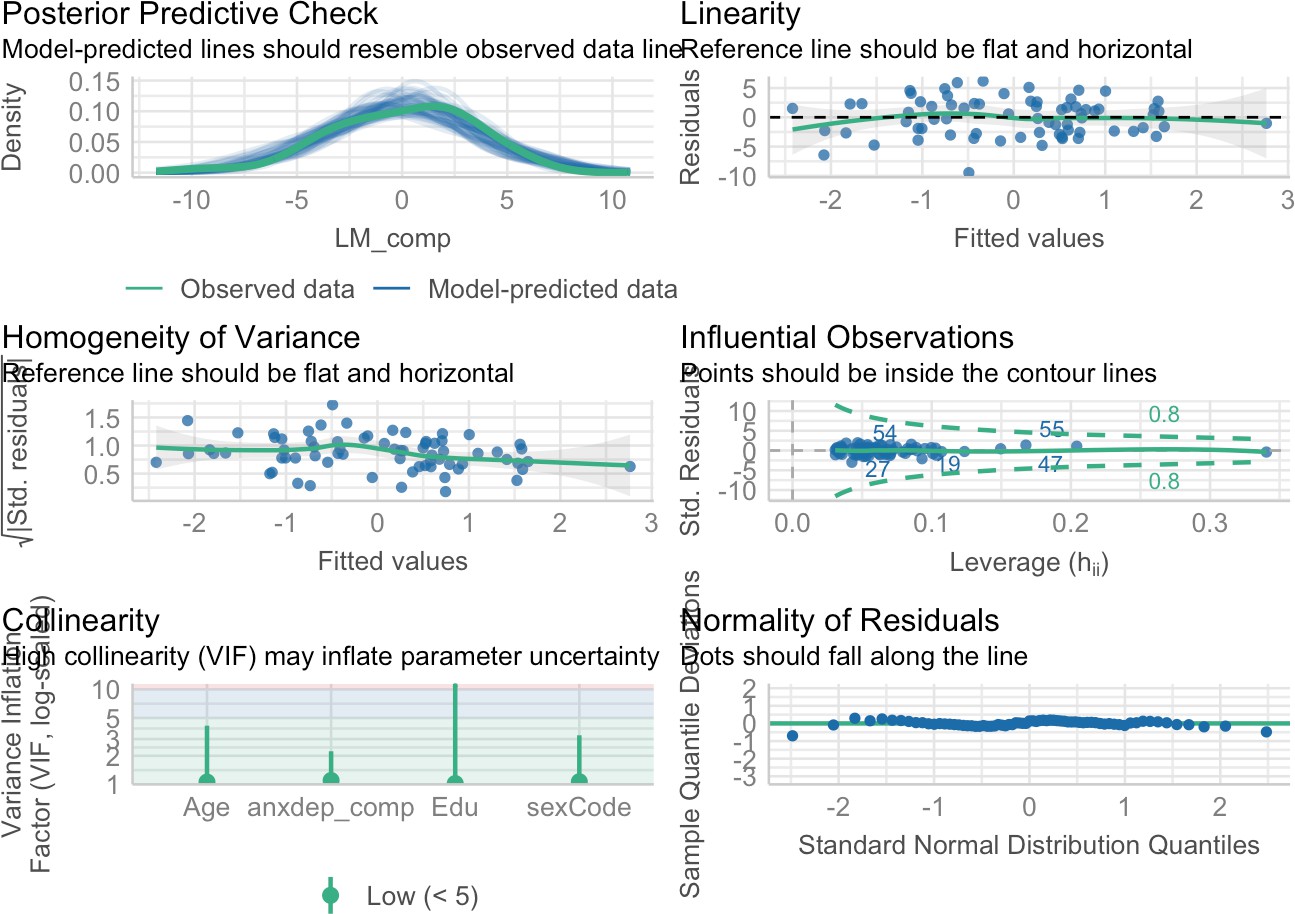


bootstrap_parameters(m.LM.anx, test = "pd")

## # Fixed Effects

##

## Parameter

##

| Coefficient |

95% CI |

pd

## (Intercept) |

## anxdep_comp |

## Age |

## sexCode |

## Edu |

8.78 | [ 0.42, 17.62] | 98.10%

-1.56e-03 | [-0.29, 0.22] | 50.60%

-0.16 | [-0.29, -0.03] | 99.40%

-0.65 | [-2.12, 0.83] | 81.00%

0.14 | [-0.01, 0.34] | 96.60%

*#relationship between cognitive function and self-reported memory*

m.metamem.cog.anx <- lm(data = d.cog.meta.anx, metamem_comp ~ LM_comp + anxdep_comp + Age + sexCode + Edu) check_model(m.metamem.cog.anx)


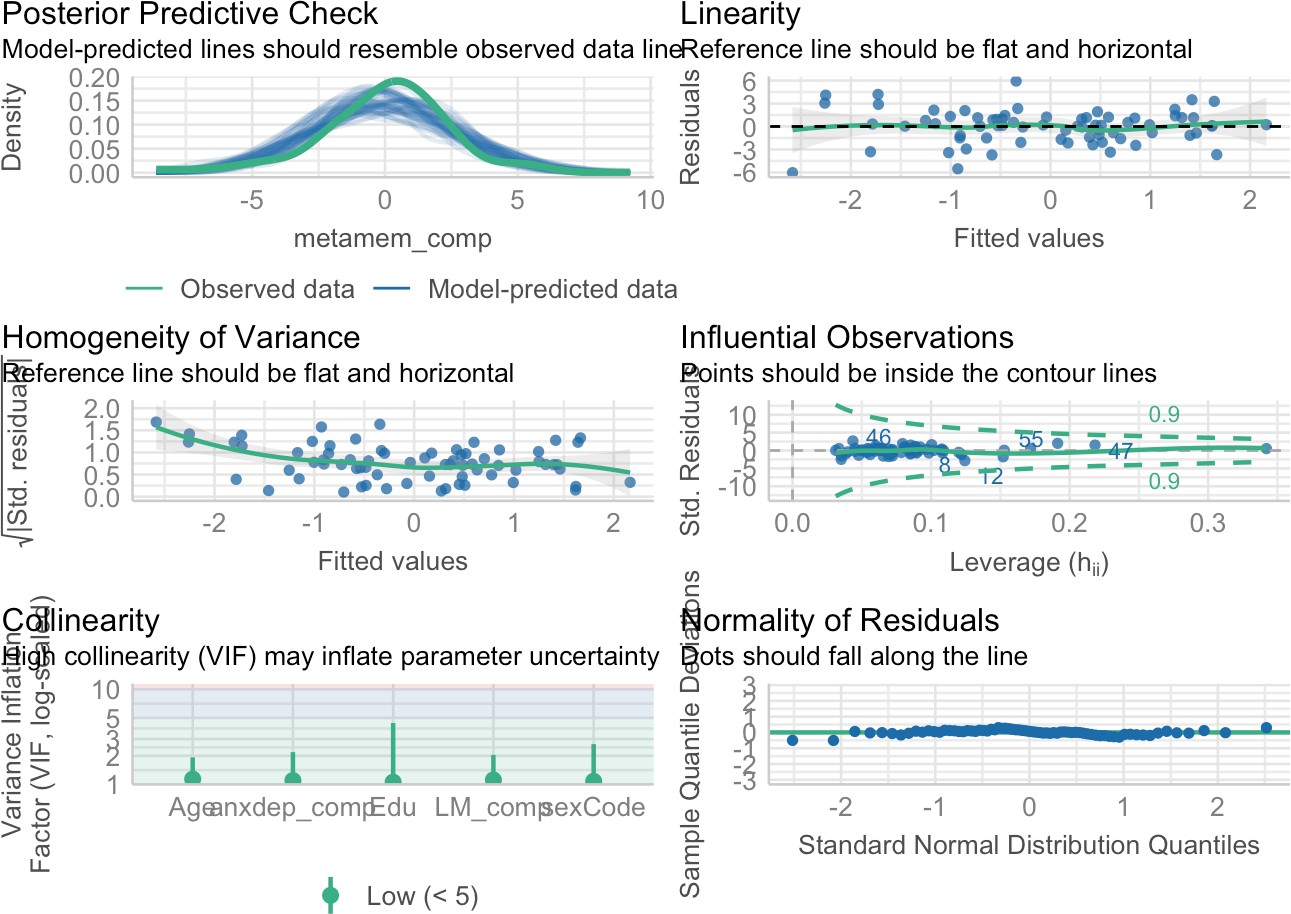


bootstrap_parameters(m.metamem.cog.anx, test = "pd")

## # Fixed Effects

##

## Parameter

##

| Coefficient |

95% CI |

pd

## (Intercept) | 0.92 | [-4.89, 6.92] | 61.40%

## LM_comp | 0.12 | [-0.04, 0.27] | 93.40%

## anxdep_comp | -0.18 | [-0.44, -0.02] | 99.20%

## Age | -0.04 | [-0.12, 0.05] | 81.00%

## sexCode | 0.98 | [ 0.01, 1.93] | 97.70%

## Edu | 0.06 | [-0.10, 0.22] | 78.30%

*#testing for relationships between different metamemory sub-scales*

m.metamem.gen.cog.anx <- lm(data = d.cog.meta.anx, Gen ~ LM_comp + anxdep_comp + Age + sexCode + Edu) check_model(m.metamem.gen.cog.anx)


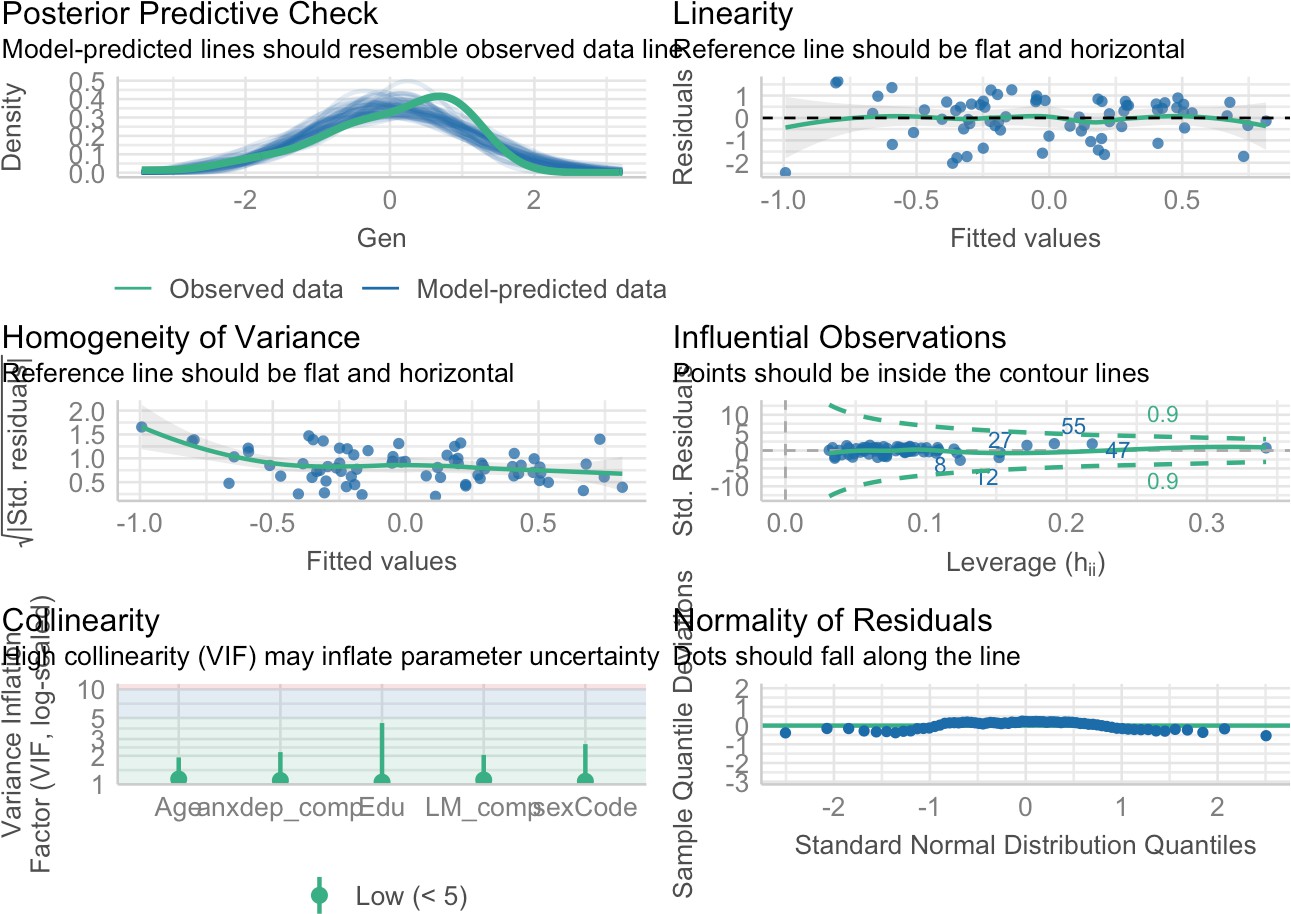


bootstrap_parameters(m.metamem.gen.cog.anx, test = "pd")

## # Fixed Effects

##

## Parameter

##

| Coefficient |

95% CI |

pd

## (Intercept) | -0.48 | [-2.95, 2.04] | 66.20%

## LM_comp | 0.06 | [ 0.00, 0.13] | 96.90%

## anxdep_comp | -0.07 | [-0.19, -0.01] | 98.60%

## Age | -1.16e-03 | [-0.04, 0.03] | 52.30%

## sexCode | 0.28 | [-0.13, 0.73] | 91.20%

## Edu | 0.02 | [-0.04, 0.09] | 79.50%

m.metamem.ability.cog.anx <- lm(data = d.cog.meta.anx, Able ~ LM_comp + anxdep_comp + Age + sexCode + Edu) check_model(m.metamem.ability.cog.anx)


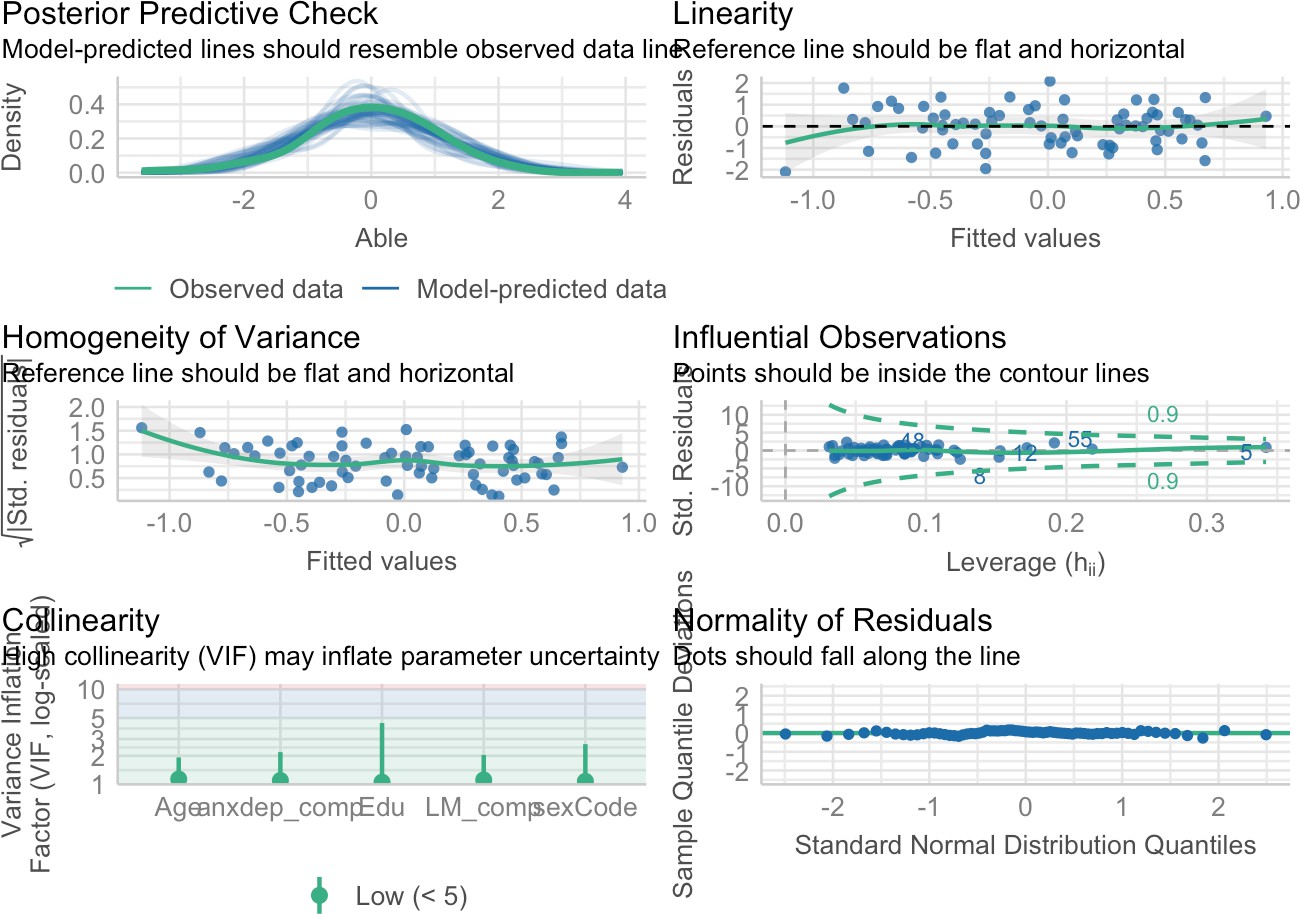


bootstrap_parameters(m.metamem.ability.cog.anx, test = "pd"

## # Fixed Effects

##

## Parameter

##

| Coefficient |

95% CI |

pd

## (Intercept) | 1.06 | [-1.89, 4.24] | 76.40%

## LM_comp | 0.06 | [ 0.01, 0.12] | 98.40%

## anxdep_comp | -0.08 | [-0.17, -0.02] | 99.40%

## Age | -0.02 | [-0.07, 0.01] | 88.20%

## sexCode | 0.16 | [-0.30, 0.60] | 75.20%

## Edu | 0.04 | [-0.03, 0.09] | 87.10%

m.metamem.strat.cog.anx <- lm(data = d.cog.meta.anx, Strat ~ LM_comp + anxdep_comp + Age + sexCode + Edu) check_model(m.metamem.strat.cog.anx)


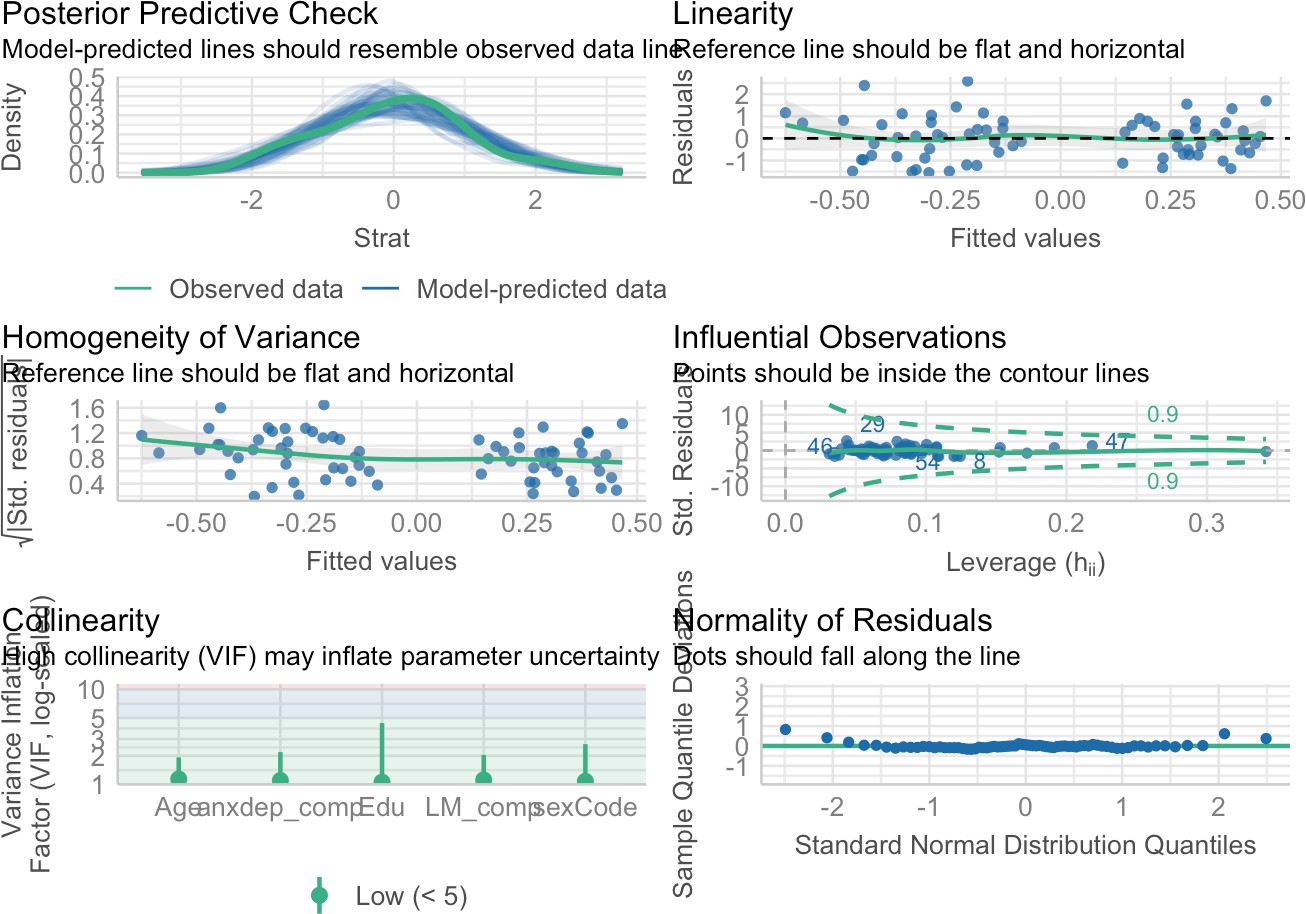


bootstrap_parameters(m.metamem.strat.cog.anx, test = "pd")

## # Fixed Effects

##

## Parameter

##

| Coefficient |

95% CI |

pd

## (Intercept) |

## LM_comp | ## anxdep_comp |

## Age |

## sexCode |

## Edu |

0.30 | [-2.80, 3.75] | 57.60%

-5.77e-03 | [-0.08, 0.07] | 55.80%

-0.03 | [-0.12, 0.04] | 78.00%

-9.87e-03 | [-0.06, 0.04] | 65.10%

0.58 | [ 0.11, 1.04] | 99.10%

-6.03e-04 | [-0.05, 0.07] | 51.10%

*#Braak staging ROI groups*

BRAAK1 = c("L_entorhinal" "R_entorhinal") BRAAK2 = c("L_hippocampus" "R_hippocampus")

*#creating data frame with relevant variables*

d.Braak1 <- d.SUVR %>% filter(label %**in**% BRAAK1) %>% group_by(BABSID, PVC) %>% summarise(mean_weighted_SUVR = (sum(num_voxels*SUVR)/sum(num_voxels))) %>% mutate(ROI = "Braak1") %>% fil

ter(PVC == "PVC") %>% select(-PVC)

d.Braak2 <- d.SUVR %>% filter(label %**in**% BRAAK2) %>% group_by(BABSID, PVC) %>% summarise(mean_weighted_SUVR = (sum(num_voxels*SUVR)/sum(num_voxels))) %>% mutate(ROI = "Braak2") %>% fil

ter(PVC == "PVC") %>% select(-PVC) d.Tau <- rbind(d.Braak1, d.Braak2) %>%

pivot_wider(id_cols = "BABSID",names_from = "ROI", values_from = "mean_weighted_SUVR") %>% ungroup() %>% mutate(Subject = parse_number(BABSID)) %>% select(-BABSID)

*#subcortical volume estimates*

d.MRI.vol <- read_csv(MRI.vol.path) %>%

mutate(Subject = parse_number(BABSID)) %>% select(-BABSID) %>%

select(Subject, ends_with("Hippocampus"), ends_with("Amygdala"), ends_with("IntraCranialVol")) %>% rename(total_ICV = EstimatedTotalIntraCranialVol, HC_R = `Right-Hippocampus`, HC_L = `Left-Hippocampus`,

amygdala_R = `Right-Amygdala`, amygdala_L = `Left-Amygdala`)

*#calculating slopes for volume adjustment ##Hippocamppus*

m.HC.R <- lm(data = d.MRI.vol, HC_R ~ total_ICV)

m.HC.R.coef <- m.HC.R$coefficients %>% as_data_frame() %>% slice(2) %>% as.numeric() m.HC.L <- lm(data = d.MRI.vol, HC_L ~ total_ICV)

m.HC.L.coef <- m.HC.L$coefficients %>% as_data_frame() %>% slice(2) %>% as.numeric()

*#adjusted subcortical volume estimates*

d.MRI.vol.adj <- d.MRI.vol %>% mutate(HC_L_slope = m.HC.L.coef,

HC_R_slope = m.HC.R.coef, mean_total_ICV = mean(total_ICV), ICV_diff = total_ICV - mean_total_ICV,

HC_L_vol_adj = HC_L - (HC_L_slope*ICV_diff), HC_R_vol_adj = HC_R - (HC_R_slope*ICV_diff), HC_bilateral_sum_adj = HC_L_vol_adj + HC_R_vol_adj, HC_bilateral_mean_adj = (HC_L_vol_adj + HC_R_vol_adj)/2)

*#cortical volume estimates*

d.LH.cort.vol <- read_csv(MRI.cort.vol.L.path) %>% mutate(Subject = parse_number(BABSID)) %>% select(-BABSID) %>% select(Subject, starts_with("lh_entorhinal")) %>%

rename(EC_L = lh_entorhinal_volume) d.RH.cort.vol <- read_csv(MRI.cort.vol.R.path) %>%

mutate(Subject = parse_number(BABSID)) %>% select(-BABSID) %>% select(Subject, starts_with("rh_entorhinal")) %>%

rename(EC_R = rh_entorhinal_volume)

d.MRI.all.vol.adj <- d.LH.cort.vol %>% inner_join(d.RH.cort.vol) %>% inner_join(d.MRI.vol.adj, by = "Subjec t")

*#calculating slopes for volume adjustment ##Hippocamppus*

m.ERC.R <- lm(data = d.MRI.all.vol.adj, EC_R ~ total_ICV)

m.ERC.R.coef <- m.ERC.R$coefficients %>% as_data_frame() %>% slice(2) %>% as.numeric() m.ERC.L <- lm(data = d.MRI.all.vol.adj, EC_L ~ total_ICV)

m.ERC.L.coef <- m.ERC.L$coefficients %>% as_data_frame() %>% slice(2) %>% as.numeric() d.MRI.all.vol.adj <- d.MRI.all.vol.adj %>%

mutate(ERC_L_slope = m.ERC.L.coef, ERC_R_slope = m.ERC.R.coef,

ERC_L_vol_adj = EC_L - (ERC_L_slope*ICV_diff), ERC_R_vol_adj = EC_R - (ERC_R_slope*ICV_diff), ERC_bilateral_sum_adj = ERC_L_vol_adj + ERC_R_vol_adj, ERC_bilateral_mean_adj = (ERC_L_vol_adj + ERC_R_vol_adj)/2)

# Characterizing the imaging data used in the present study

# To better contextualize the neuroimaging results, we are providing visualizations of the regions of interest used in the present study.

#

#

Above, we have plotted both the left and right entorhinal cortex and hippocampus used in our study. In this plot, masks for the entorhinal cortex (red) and hippocampus (yellow) are plotted on a participant’s T1 image.

Above, we have plotted the mean PET images with the ROIs defined by the Desikan-Killiany atlas overlaid. A) Mean PET image (grayscale) from a 67-year-old female participant from 90-110 min post-injection. B) Overlaid ROIs (multi-color) defined by the Desikan-Killiany atlas on the mean PET image shown in panel A. C) Mean PET image (gray scale) from a 67-year-old male participant from 90-110 min post-injection. D) Overlaid ROIs (multi-color) defined by the Desikan-Killiany atlas on the mean PET image shown in panel C.

Above, group averaged SUVR image with the partial volume corrected (PVC) values shown. Brighter blue values correspond to higher SUVRs. Consistent with prior work, the highest observed SUVR estimates are in the entorhinal cortex.

.

# Examining the zero-order correlations between MRI measures and age

## Overall, we find that age is negatively associated with measures of volume and thickness (caudate volume is an exception)

*#import tau data*

d.cog.meta.tau <- d.cog.meta.anx %>% inner_join(d.Tau, by = "Subject") %>% inner_join(d.MRI.all.vol.adj, by "Subject") %>%

mutate(Braak1 = scale(Braak1, center = T, scale = T), Braak2 = scale(Braak2, center = T, scale = T),

ERC_bilateral_sum_adj = scale(ERC_bilateral_sum_adj, center = T, scale = T), HC_bilateral_sum_adj = scale(HC_bilateral_sum_adj, center = T, scale = T))

*#plotting the associations between ICV-adjusted volume and age #EC*

p.ERC.vol.age <- plot(cor_test(data= d.cog.meta.tau, x = "Age", y = "ERC_bilateral_sum_adj"), smooth= aes(c olor="slateblue2")) + theme_classic() + labs(x= "Age", y="EC Volume (standardized)")

p.ERC.vol.age


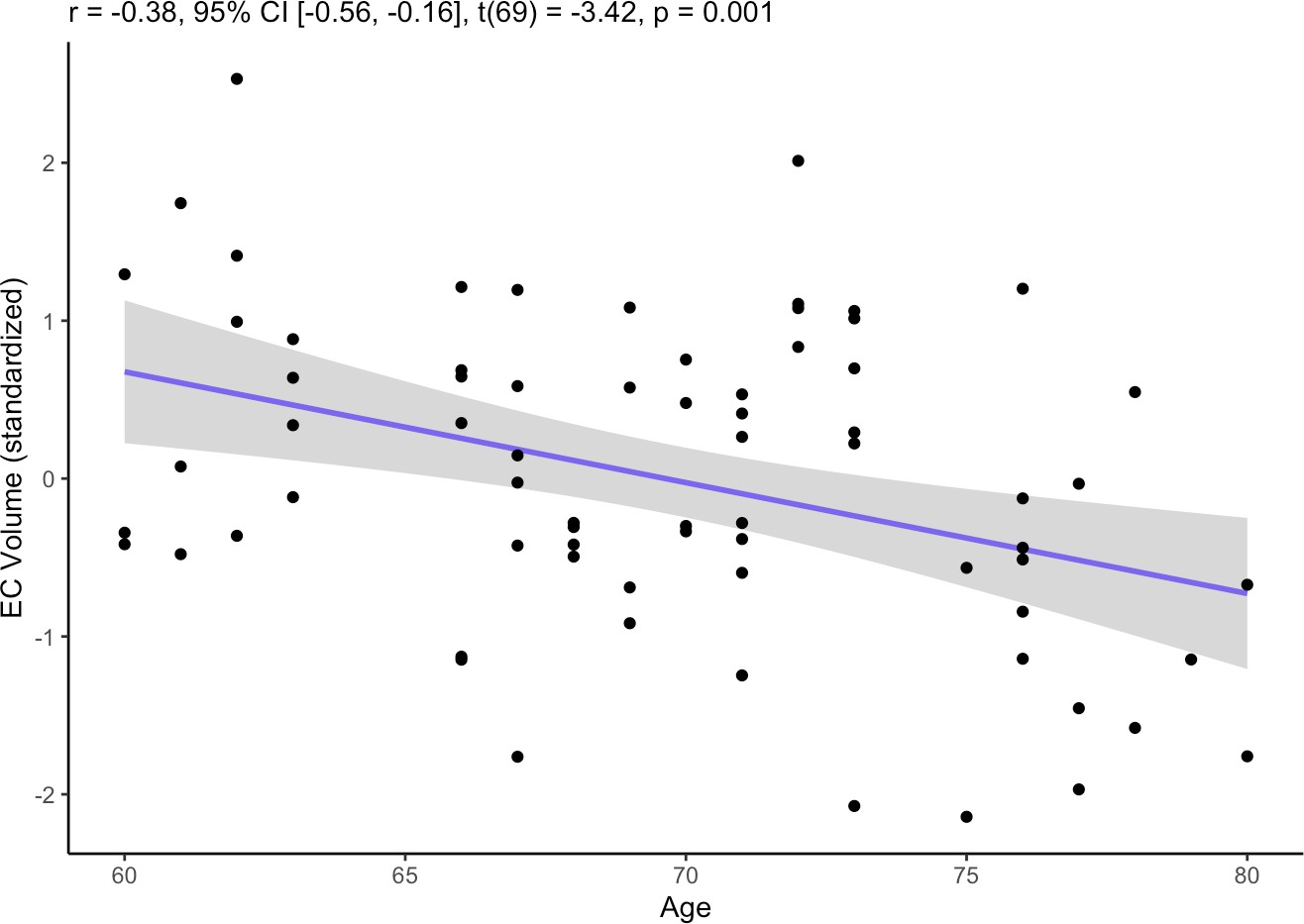


*#HC*

p.HC.vol.age <- plot(cor_test(data= d.cog.meta.tau, x = "Age", y = "HC_bilateral_sum_adj"), smooth= aes(col or="slateblue2")) + theme_classic() + labs(x= "Age", y="Hippocampal Volume (standardized)")

p.HC.vol.age


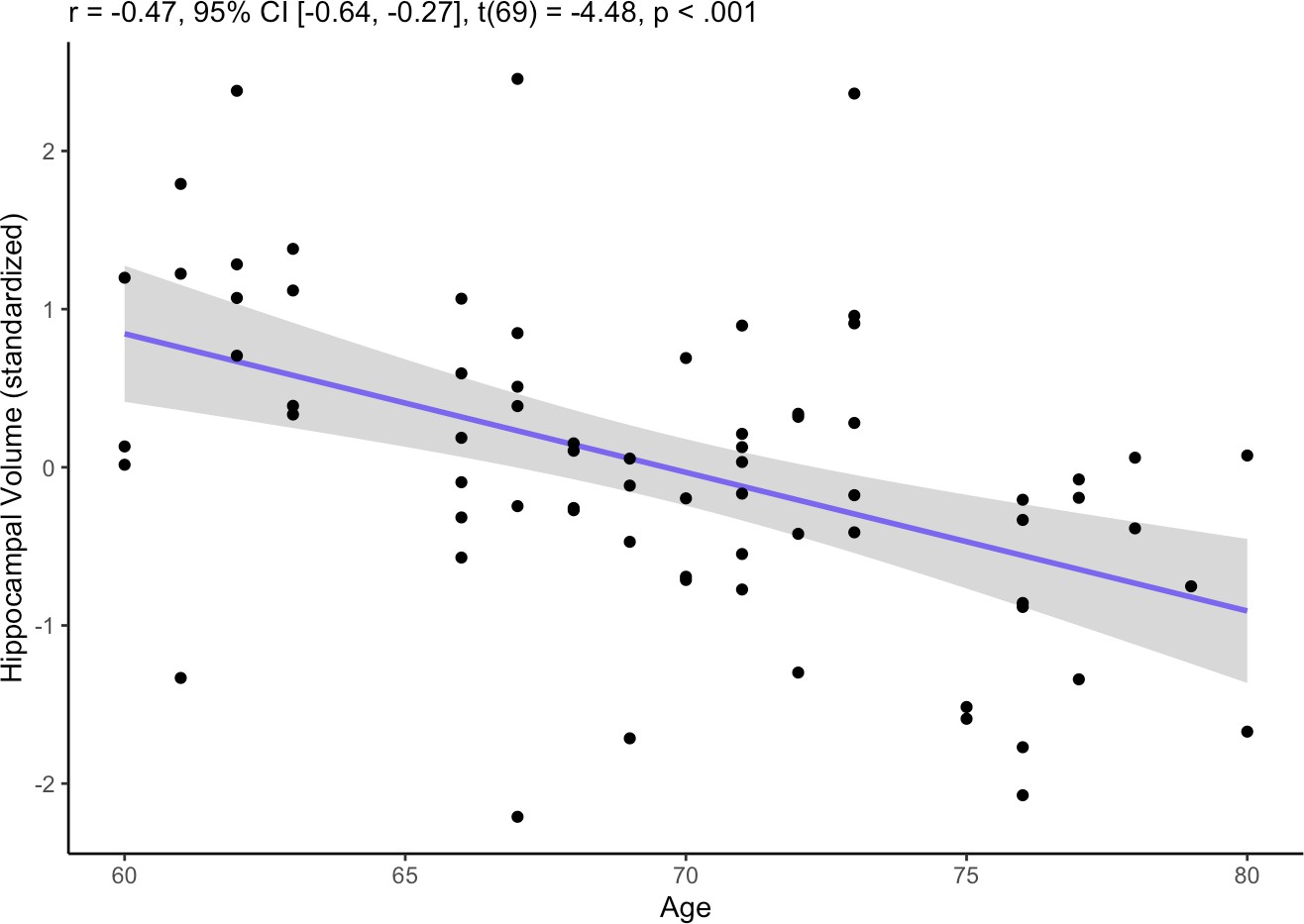


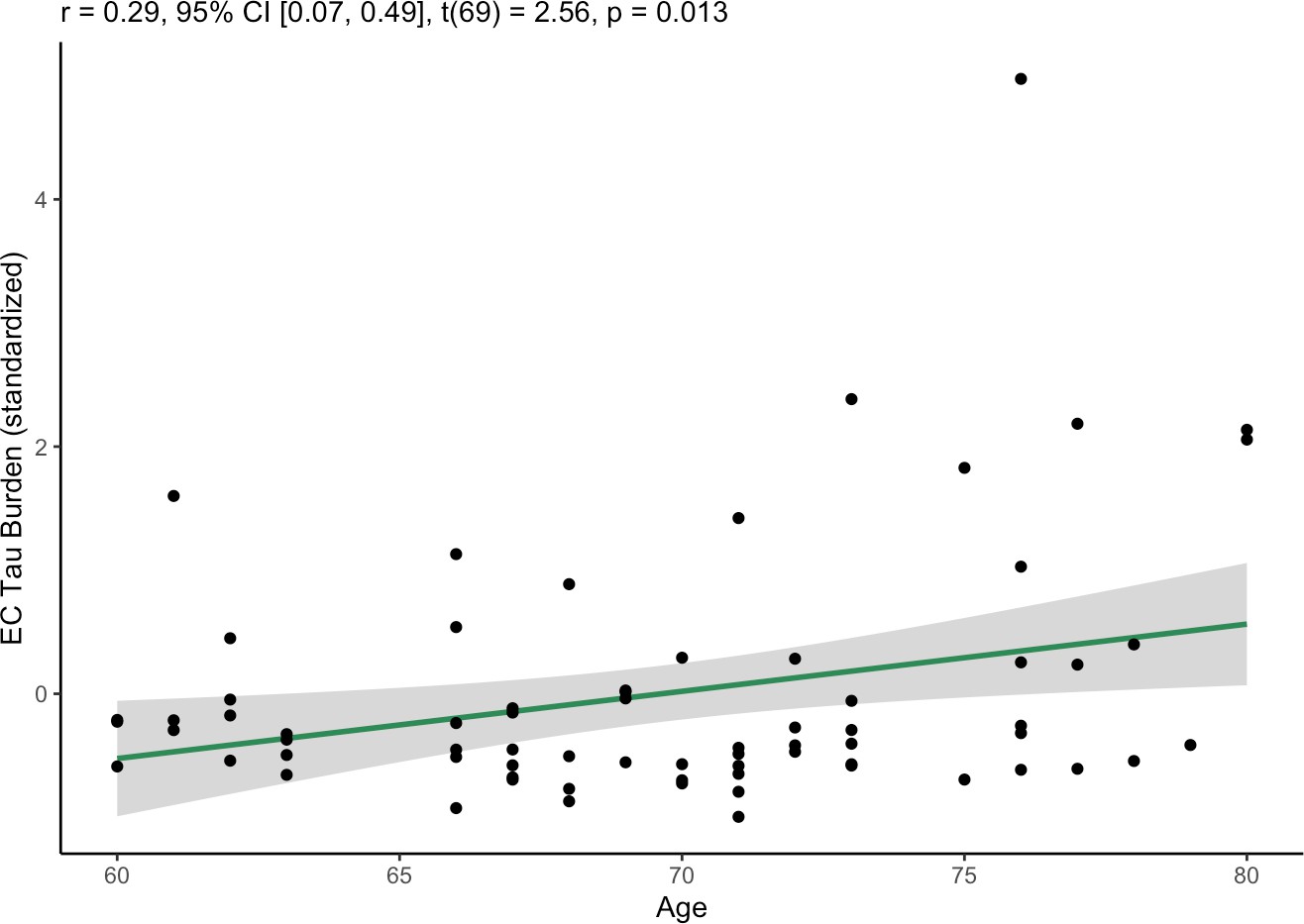


*#plotting the associations between tau burden and age #EC*

p.ERC.tau.age <- plot(cor_test(data= d.cog.meta.tau, x = "Age", y = "Braak1"), smooth= aes(color="seagree n")) + theme_classic() + labs(x= "Age", y="EC Tau Burden (standardized)")

p.ERC.tau.age

*#HC*

p.HC.tau.age <- plot(cor_test(data= d.cog.meta.tau, x = "Age", y = "Braak2"), smooth= aes(color="seagree n")) + theme_classic() + labs(x= "Age", y="Hippocampal Tau Burden (standardized)"

p.HC.tau.age


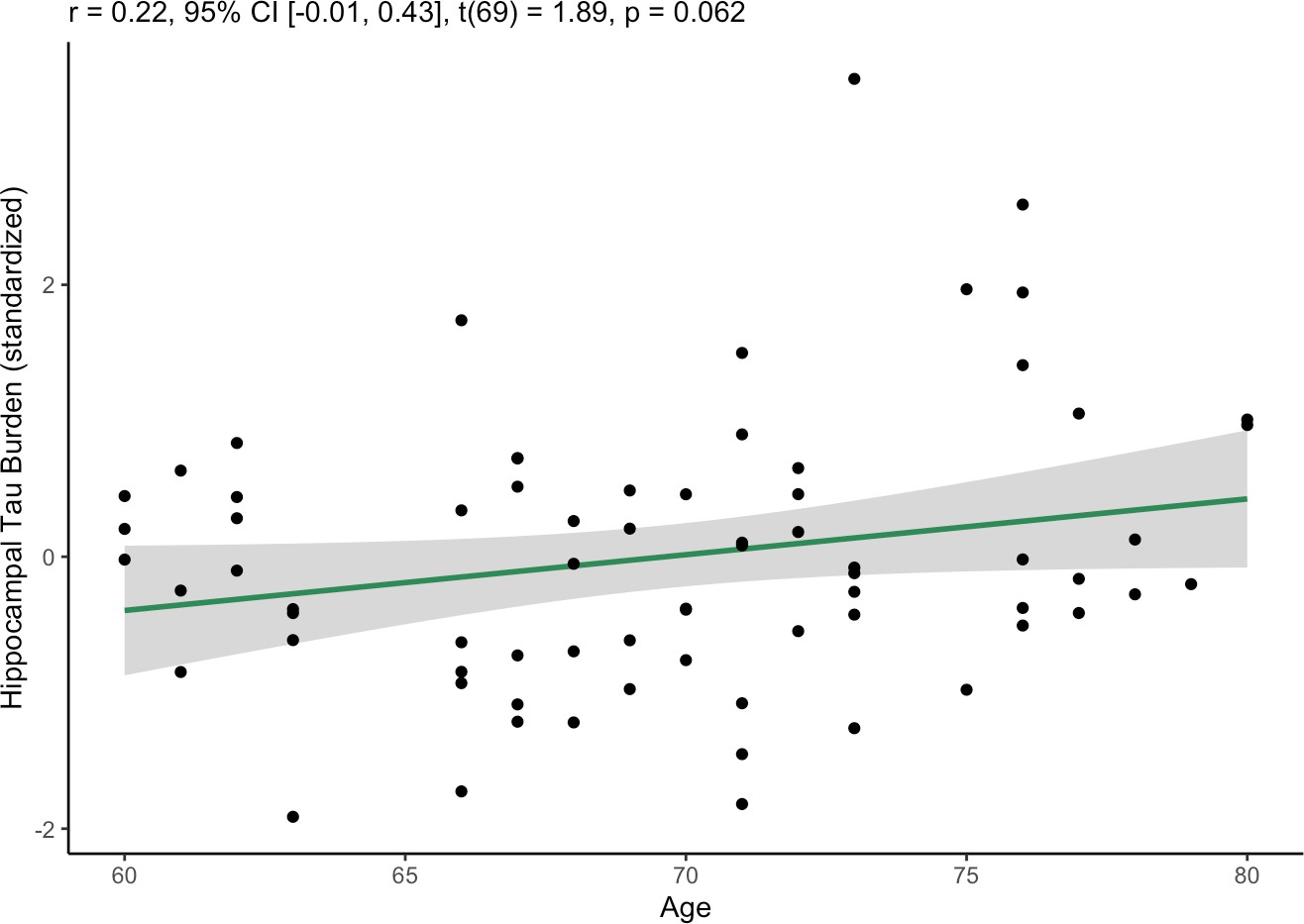


# Summarizing model diagnostics from neural analyses

*#relationship between cognitive ability and gray matter volume*

m.LM.gm.EC <- lm(data = d.cog.meta.tau, LM_comp ~ ERC_bilateral_sum_adj + Age + sexCode + Edu) check_model(m.LM.gm.EC)


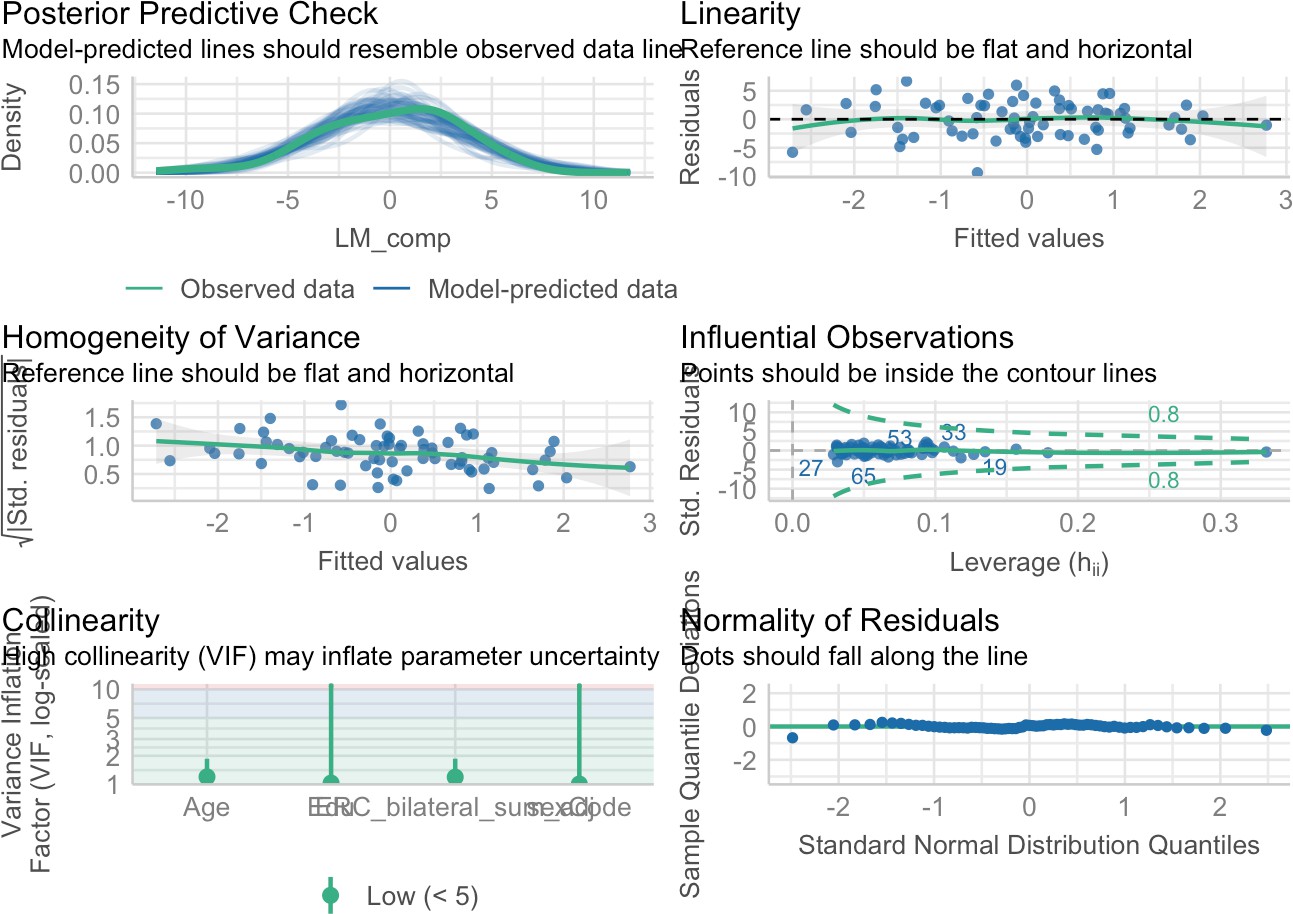


bootstrap_parameters(m.LM.gm.EC, test = "pd")

## # Fixed Effects ##

## Parameter ##

## (Intercept)

| Coefficient |

95% CI |

pd

|

## ERC_bilateral_sum_adj | ## Age |

## sexCode |

## Edu |

7.36 | [-1.29, 15.64] | 95.30%

0.44 | [-0.40, 1.31] | 85.20%

-0.14 | [-0.26, -0.01] | 98.40%

-0.61 | [-2.14, 0.91] | 78.70%

0.13 | [-0.02, 0.32] | 94.50%

m.LM.gm.HC <- lm(data = d.cog.meta.tau, LM_comp ~ HC_bilateral_sum_adj + Age + sexCode + Edu) check_model(m.LM.gm.HC)


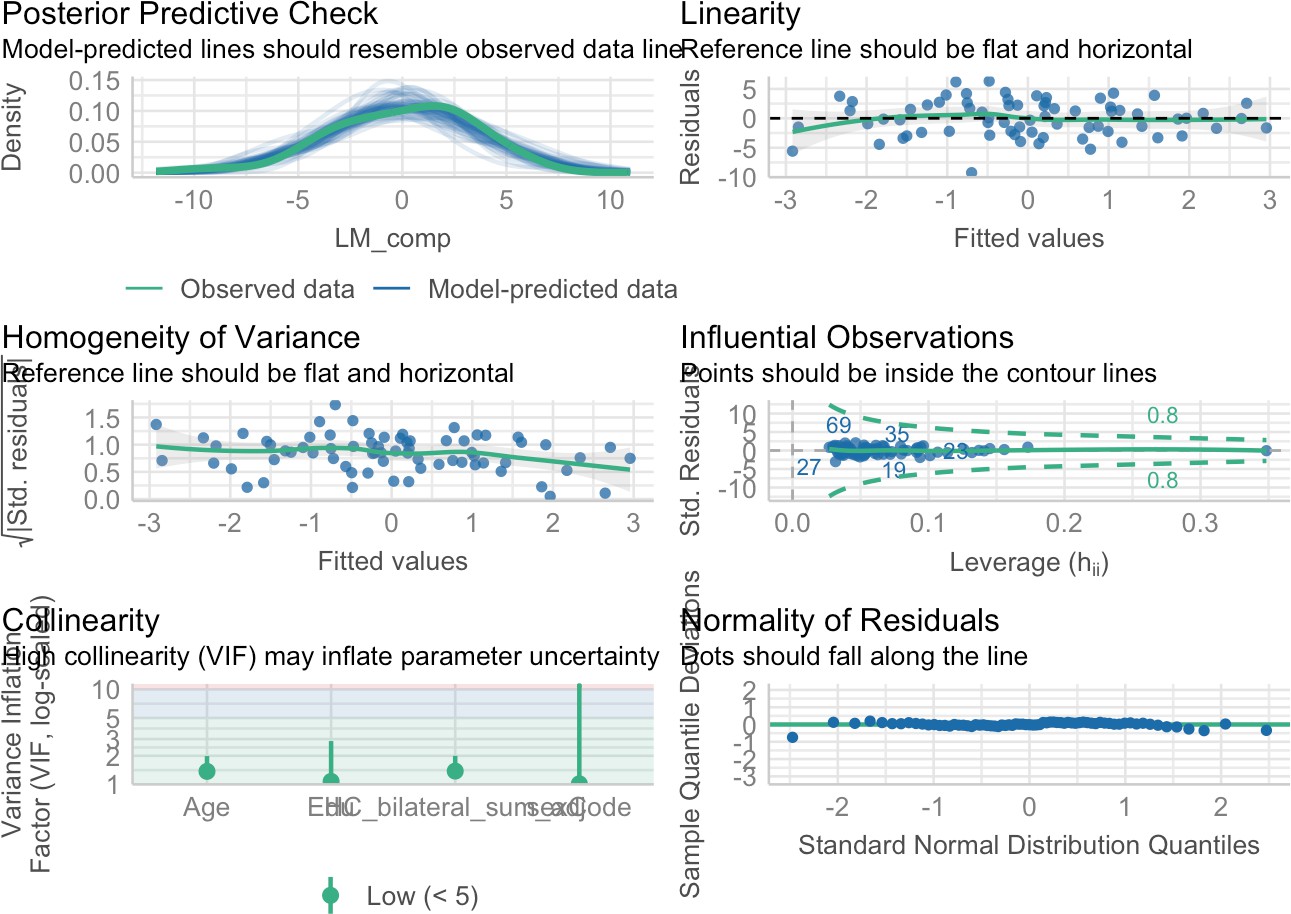


bootstrap_parameters(m.LM.gm.HC, test = "pd")

## # Fixed Effects ##

## Parameter ##

## (Intercept)

| Coefficient |

95% CI |

pd

|

3.67 | [-4.87, 12.57] | 81.20%

## HC_bilateral_sum_adj | 0.96 | [ 0.37, 1.66] | 99.50%

## Age | -0.07 | [-0.20, 0.05] | 88.60%

## sexCode | -0.79 | [-2.32, 0.66] | 85.80%

## Edu | 0.09 | [-0.08, 0.27] | 90.30%

*#relationship between cognitive ability and gray matter volume controlling for anxiety and depression sympt oms*

m.LM.gm.EC.anx <- lm(data = d.cog.meta.tau, LM_comp ~ ERC_bilateral_sum_adj + Age + sexCode + Edu + anxdep_ comp)

check_model(m.LM.gm.EC.anx)


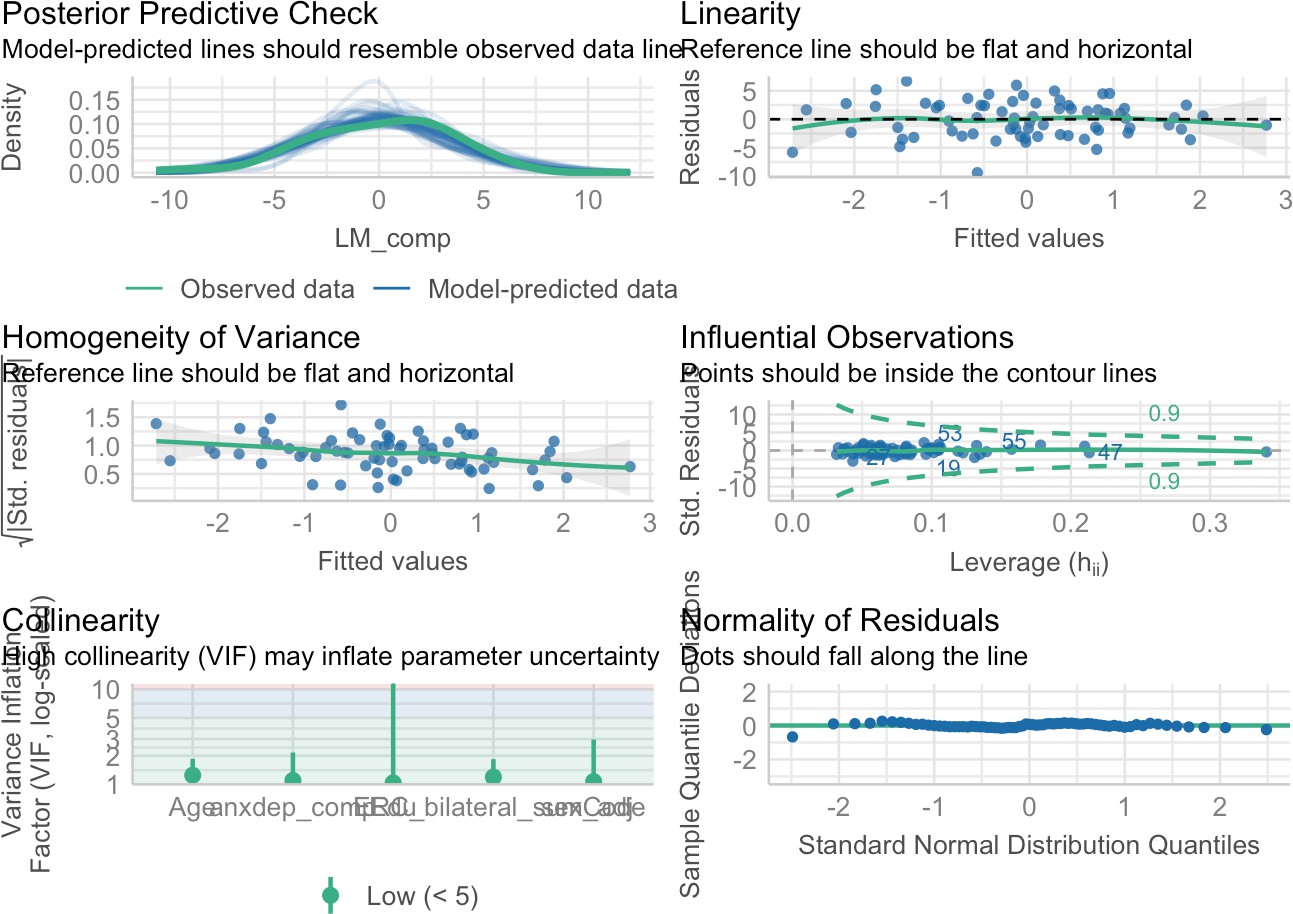


bootstrap_parameters(m.LM.gm.EC.anx, test = "pd"

## # Fixed Effects ##

## Parameter ##

## (Intercept)

| Coefficient |

95% CI |

pd

|

6.79 | [-1.48, 16.44] | 94.60%

## ERC_bilateral_sum_adj |

## Age |

## sexCode |

## Edu |

## anxdep_comp |

0.44 | [-0.40, 1.33] | 81.80%

-0.13 | [-0.27, 0.00] | 97.60%

-0.54 | [-2.06, 0.86] | 78.80%

0.13 | [-0.02, 0.32] | 95.80%

-6.79e-04 | [-0.30, 0.21] | 50.40%

m.LM.gm.HC.anx <- lm(data = d.cog.meta.tau, LM_comp ~ HC_bilateral_sum_adj + Age + sexCode + Edu + anxdep_c omp)

check_model(m.LM.gm.HC.anx)


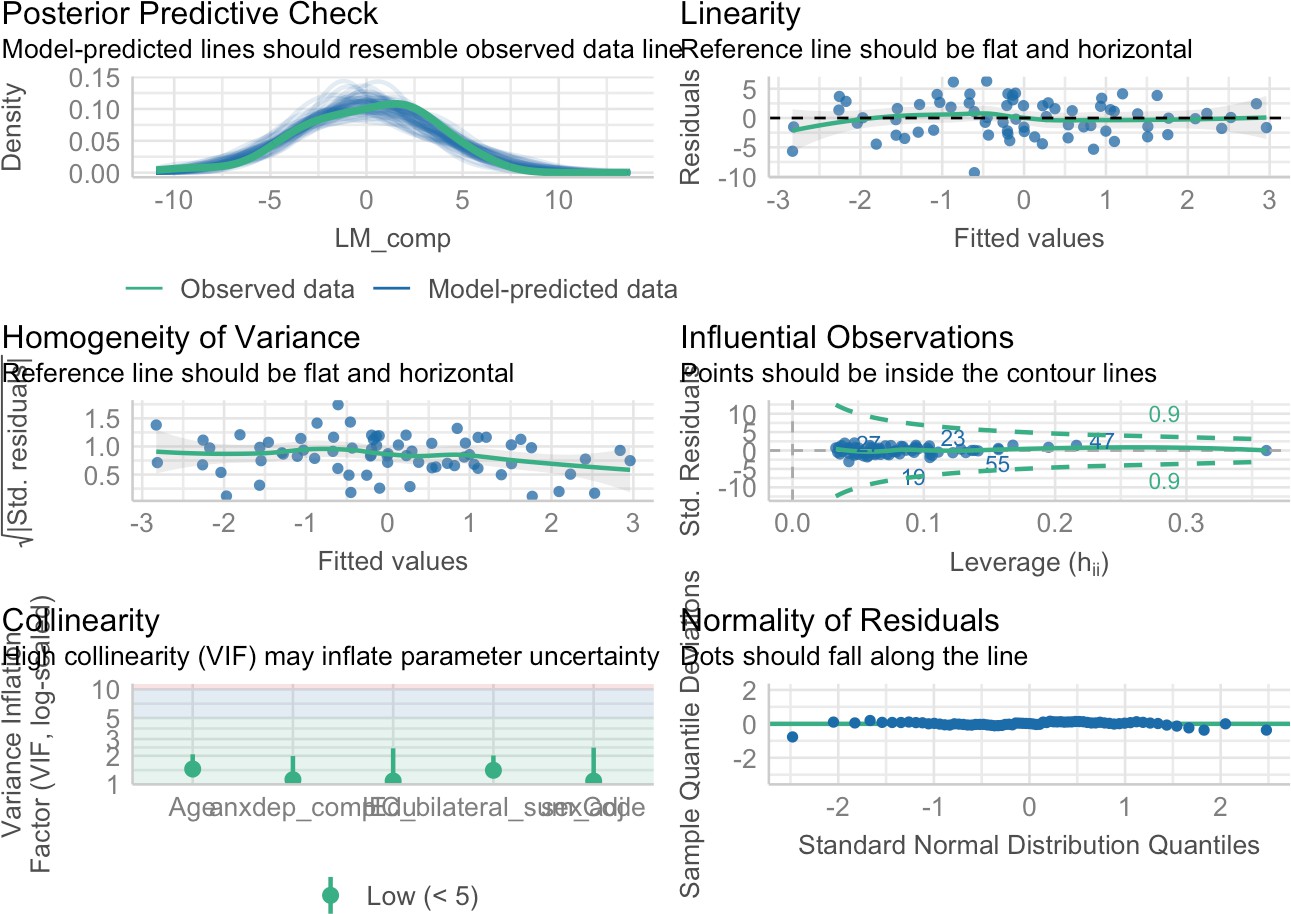


bootstrap_parameters(m.LM.gm.HC.anx, test = "pd"

## # Fixed Effects ##

## Parameter ##

## (Intercept)

| Coefficient |

95% CI |

pd

|

3.44 | [-5.49, 13.22] | 76.50%

## HC_bilateral_sum_adj | 1.01 | [ 0.28, 1.62] | 99.60%

## Age | -0.07 | [-0.22, 0.06] | 83.50%

## sexCode | -0.81 | [-2.29, 0.71] | 85.30%

## Edu | 0.09 | [-0.10, 0.28] | 87.10%

## anxdep_comp | -0.02 | [-0.34, 0.17] | 58.00%

*#relationship between metamemory and gray matter volume*

m.metamem.gm.EC <- lm(data = d.cog.meta.tau, metamem_comp ~ ERC_bilateral_sum_adj + Age + sexCode + Edu) check_model(m.metamem.gm.EC)


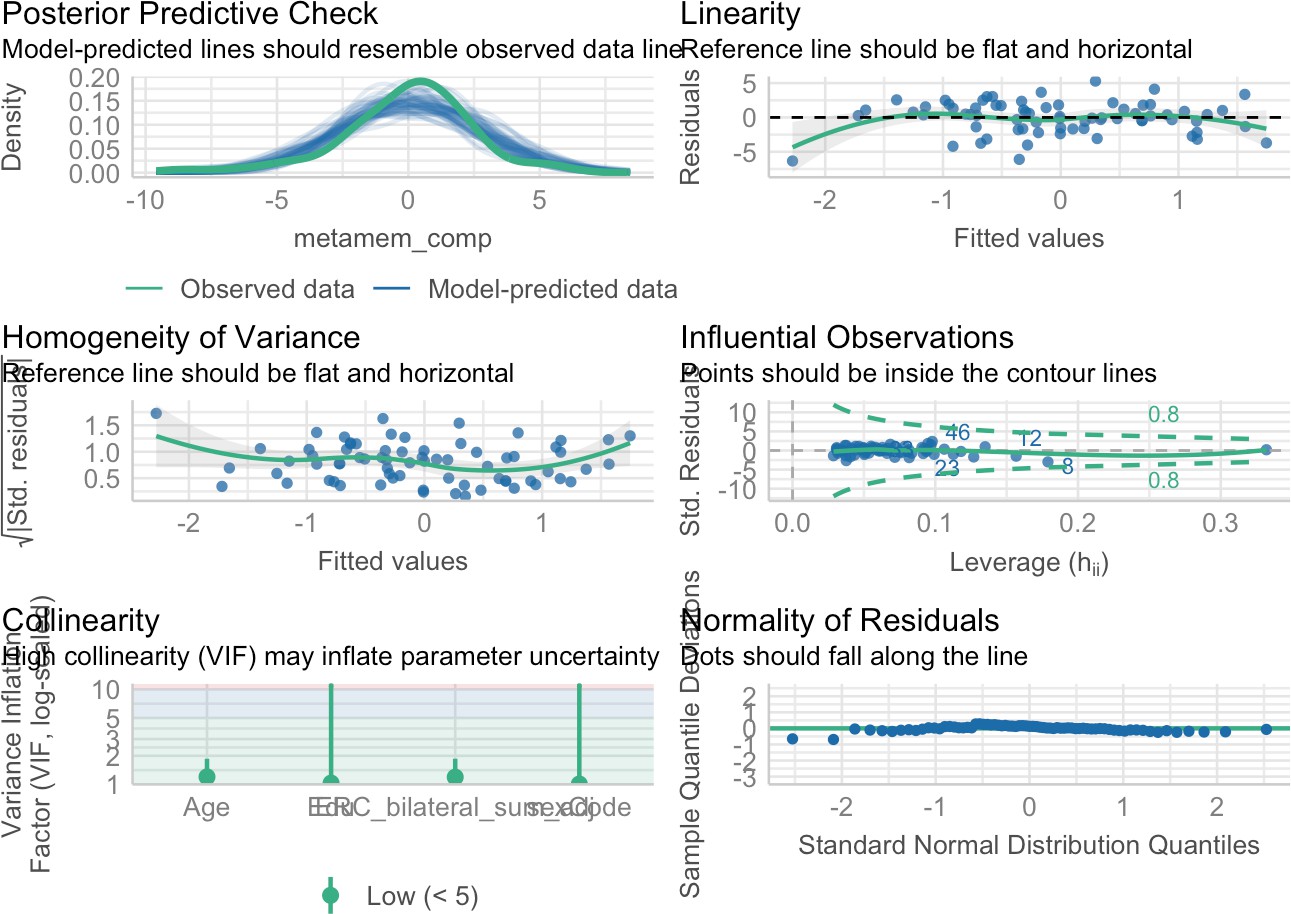


bootstrap_parameters(m.metamem.gm.EC,test = "pd"

## # Fixed Effects ##

## Parameter ##

## (Intercept)

| Coefficient |

95% CI |

pd

|

4.57 | [-2.26, 11.65] | 87.90%

## ERC_bilateral_sum_adj | -0.31 | [-1.12, 0.54] | 77.70%

## Age | -0.10 | [-0.21, 0.01] | 96.80%

## sexCode | 1.23 | [ 0.16, 2.28] | 98.30%

## Edu | 0.09 | [-0.07, 0.28] | 86.80%

m.metamem.gm.HC <- lm(data = d.cog.meta.tau, metamem_comp ~ HC_bilateral_sum_adj + Age + sexCode + Edu) check_model(m.metamem.gm.HC)


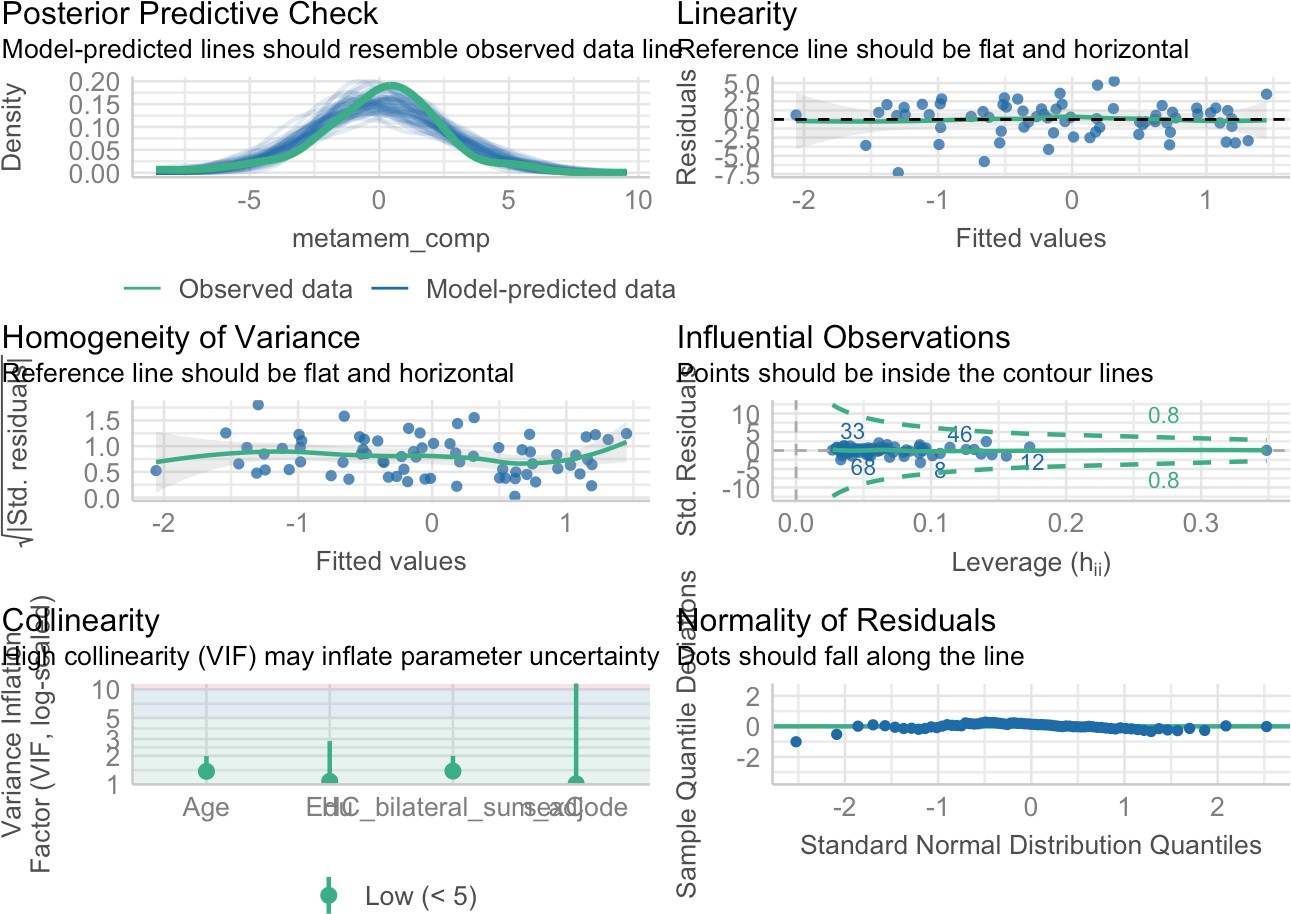


bootstrap_parameters(m.metamem.gm.HC, test = "pd")

## # Fixed Effects ##

## Parameter ##

## (Intercept)

| Coefficient |

95% CI |

pd

|

4.85 | [-3.47, 14.52] | 85.80%

## HC_bilateral_sum_adj | -0.31 | [-1.15, 0.45] | 77.80%

## Age | -0.10 | [-0.24, 0.02] | 95.00%

## sexCode | 1.27 | [ 0.18, 2.35] | 99.10%

## Edu | 0.09 | [-0.06, 0.31] | 89.40%

*#relationship between metamemory and gray matter volume controlling for anxiety and depression symptoms*

m.metamem.gm.EC.anx <- lm(data = d.cog.meta.tau, metamem_comp ~ ERC_bilateral_sum_adj + Age + sexCode + Edu

+ anxdep_comp) check_model(m.metamem.gm.EC.anx)


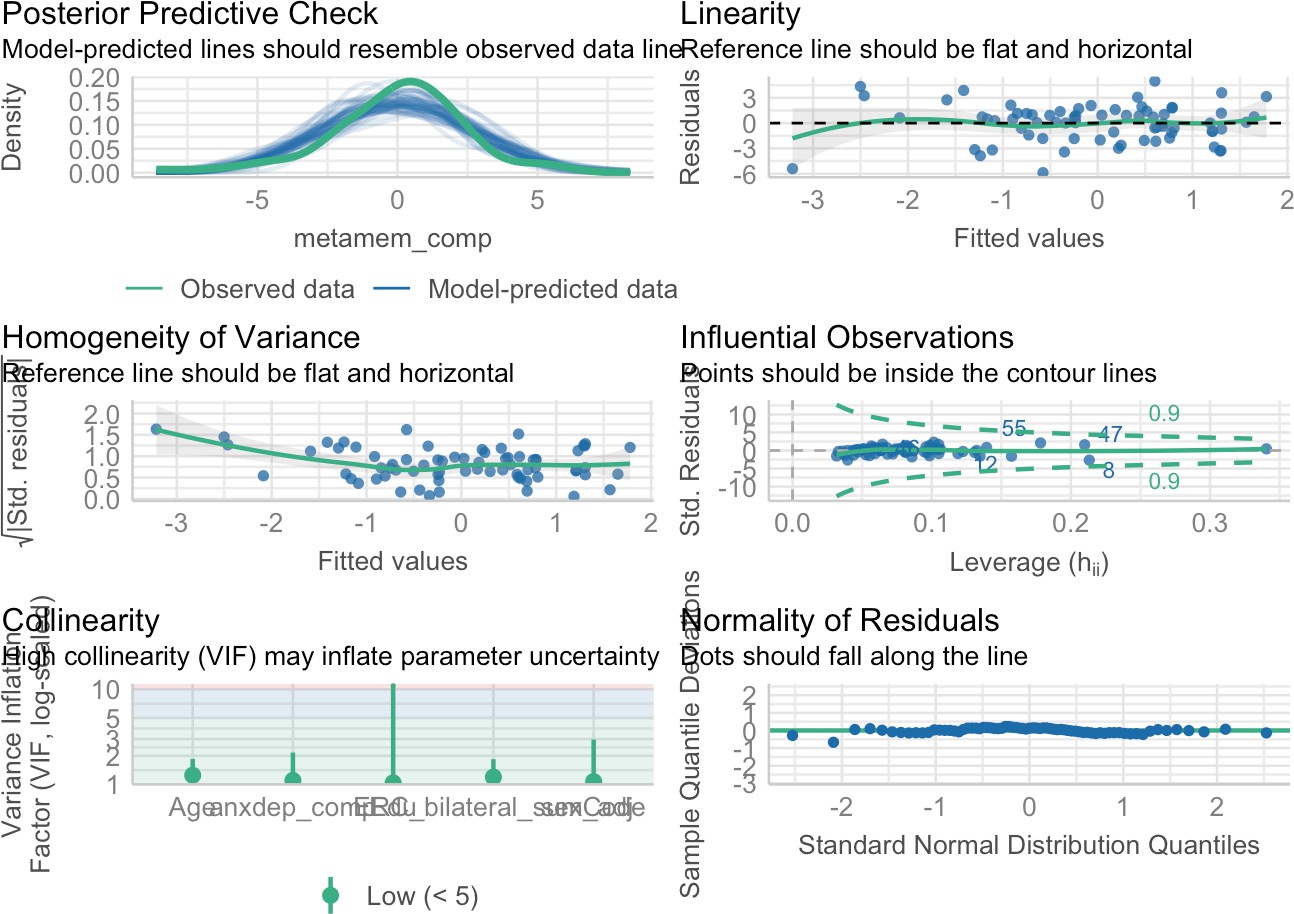


bootstrap_parameters(m.metamem.gm.EC.anx, test = "pd")

## # Fixed Effects ##

## Parameter ##

## (Intercept)

| Coefficient |

95% CI |

pd

|

3.16 | [-4.15, 10.92] | 79.60%

## ERC_bilateral_sum_adj |

## Age

## sexCode ## Edu

## anxdep_comp

|

|

|

|

-0.28 | [-0.98, 0.48] | 77.80%

-0.07 | [-0.18, 0.03] | 91.30%

0.91 | [-0.05, 1.89] | 96.70%

0.08 | [-0.08, 0.24] | 84.80%

-0.18 | [-0.46, -0.02] | 98.40%

m.metamem.gm.HC.anx <- lm(data = d.cog.meta.tau, metamem_comp ~ HC_bilateral_sum_adj + Age + sexCode + Edu

+ anxdep_comp) check_model(m.metamem.gm.HC.anx)


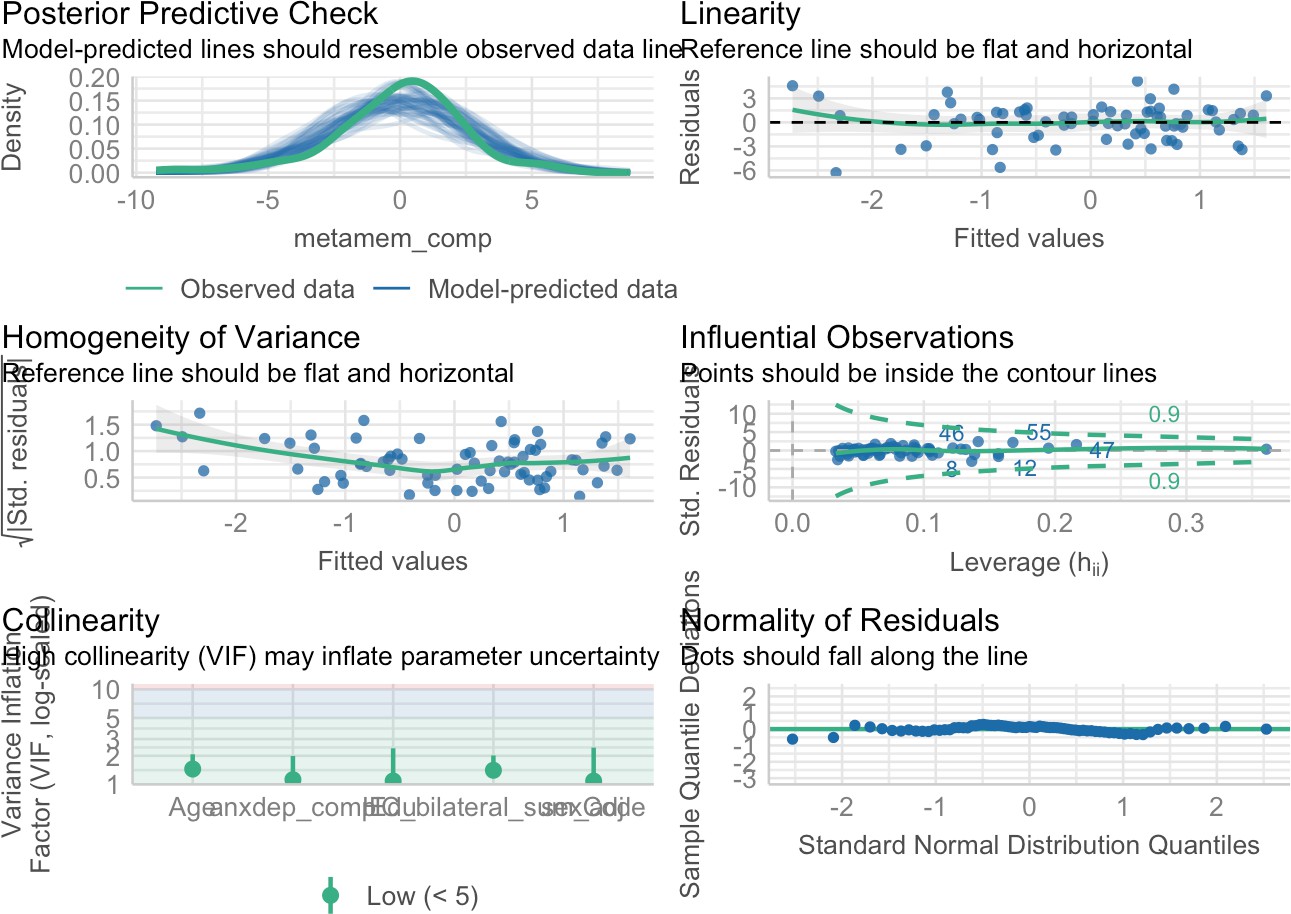


bootstrap_parameters(m.metamem.gm.HC.anx, test = "pd")

## # Fixed Effects ##

## Parameter ##

## (Intercept)

| Coefficient |

95% CI |

pd

|

2.87 | [-5.93, 11.25] | 75.30%

## HC_bilateral_sum_adj |

## Age

## sexCode ## Edu

## anxdep_comp

|

|

|

|

-0.19 | [-0.92, 0.57] | 68.20%

-0.07 | [-0.19, 0.05] | 86.50%

0.99 | [ 0.00, 1.92] | 97.50%

0.09 | [-0.06, 0.23] | 88.20%

-0.18 | [-0.43, -0.02] | 98.50%

*#relationship between cognitive ability and tau burden*

m.LM.tau.EC <- lm(data = d.cog.meta.tau, LM_comp ~ ERC_bilateral_sum_adj + Braak1 + Age + sexCode + Edu) check_model(m.LM.tau.EC)


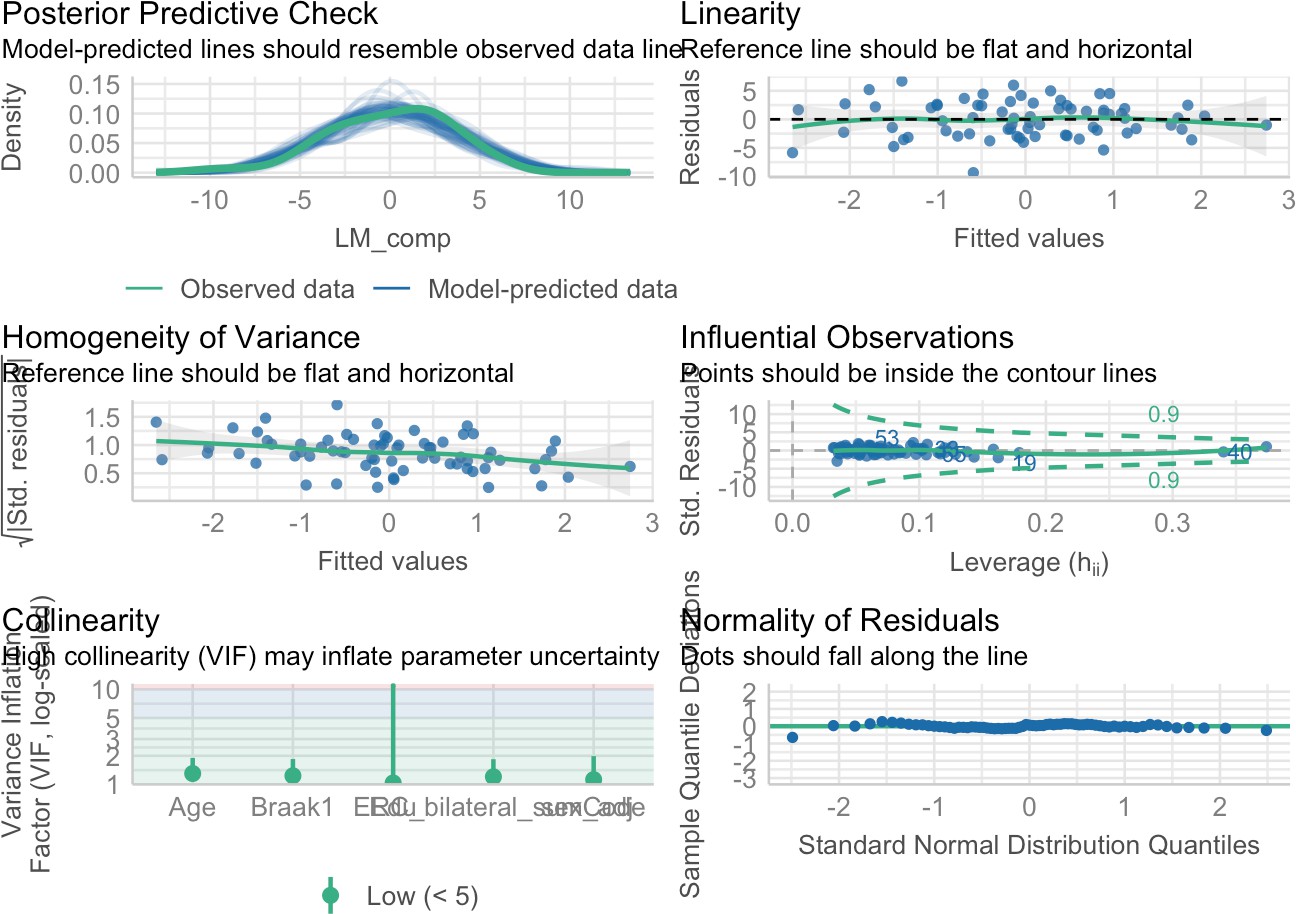


bootstrap_parameters(m.LM.tau.EC, test = "pd"

## # Fixed Effects ##

## Parameter ##

## (Intercept)

| Coefficient |

95% CI |

pd

|

## ERC_bilateral_sum_adj | ## Braak1 |

## Age |

## sexCode |

## Edu |

7.45 | [-1.20, 16.06] | 95.60%

0.46 | [-0.43, 1.39] | 85.10%

0.07 | [-1.07, 0.89] | 57.20%

-0.14 | [-0.26, -0.01] | 97.90%

-0.60 | [-2.15, 0.89] | 77.30%

0.13 | [-0.02, 0.33] | 95.50%

m.LM.tau.HC <- lm(data = d.cog.meta.tau, LM_comp ~ HC_bilateral_sum_adj + Braak2 + Age + sexCode + Edu) check_model(m.LM.tau.HC)


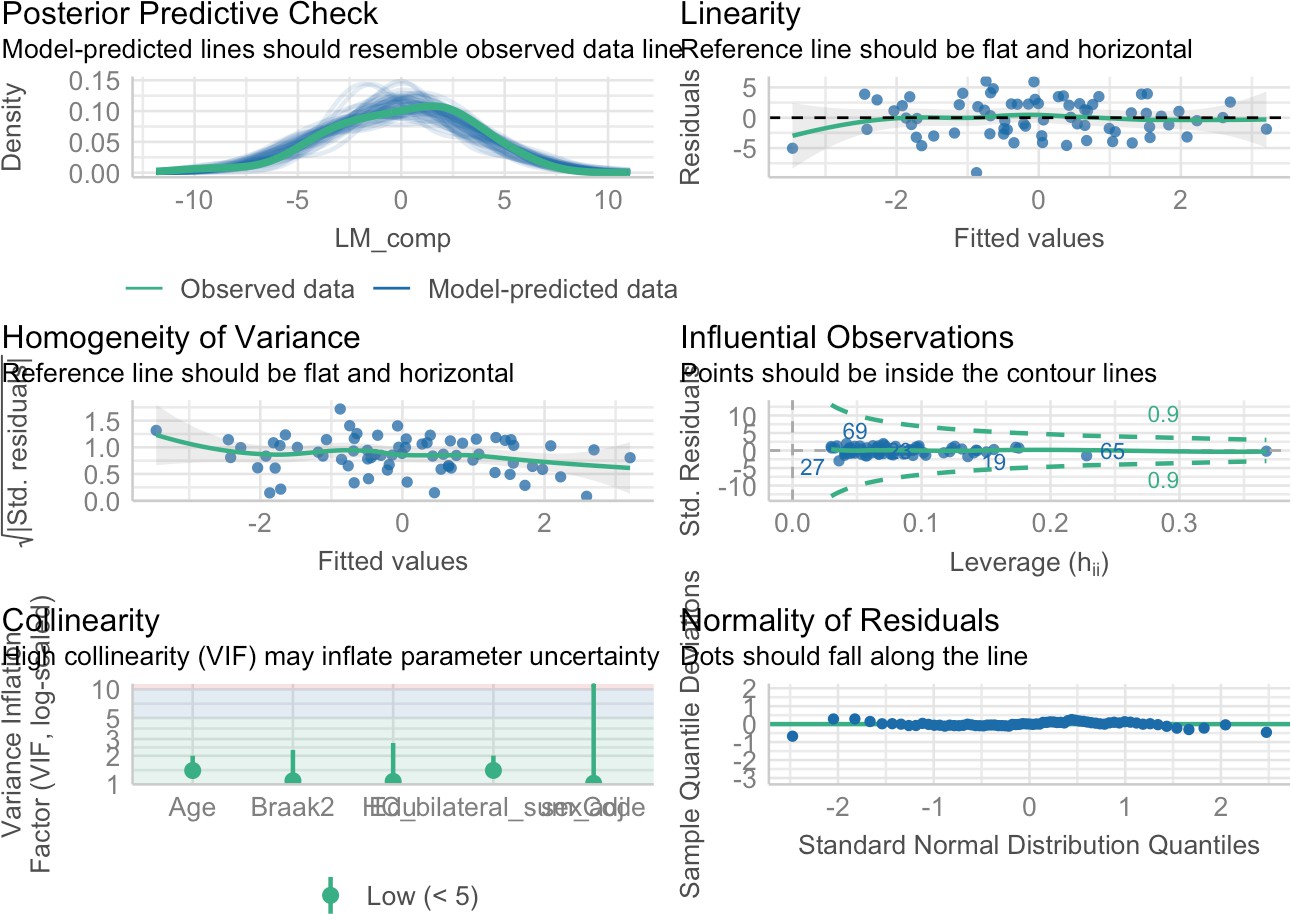


bootstrap_parameters(m.LM.tau.HC, test = "pd"

## # Fixed Effects ##

## Parameter ##

## (Intercept)

| Coefficient |

95% CI |

pd

|

3.06 | [-6.05, 12.00] | 73.80%

## HC_bilateral_sum_adj | 0.92 | [ 0.24, 1.64] | 99.90%

## Braak2 | -0.37 | [-1.12, 0.54] | 78.70%

## Age | -0.06 | [-0.19, 0.07] | 83.10%

## sexCode | -0.84 | [-2.40, 0.62] | 87.50%

## Edu | 0.09 | [-0.07, 0.27] | 89.30%

*#relationship between self-reported metacognition and tau*

m.metamem.tau.braak1 <- lm(data = d.cog.meta.tau, metamem_comp ~ LM_comp + Braak1*anxdep_comp + ERC_bilater al_sum_adj + Age + sexCode + Edu)

check_model(m.metamem.tau.braak1)


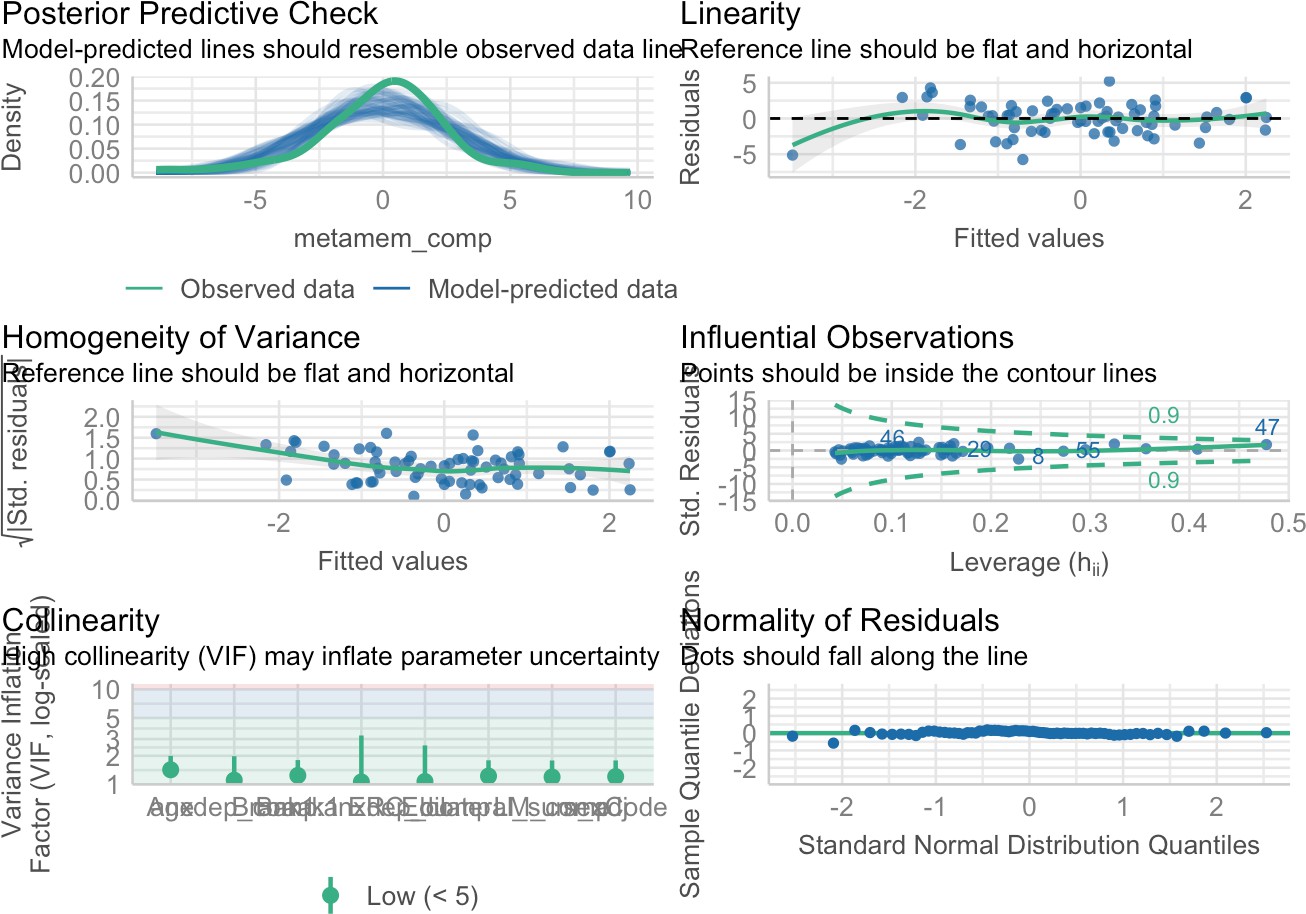


bootstrap_parameters(m.metamem.tau.braak1, test = "pd")

| ## | # Fixed Effects |  | | | | | | |
| --- | --- | --- | --- | --- | --- | --- | --- | --- |
| ## |  |  |  |  |  |  |  |  |
| ## | Parameter | \| | Coefficient | \| |  | 95% CI | \| | pd |
| ## |  |  |  |  |  |  |  |  |
| ## | (Intercept) | \| | 2.32 | \| | [-5.15, | 9.66] | \| | 71.00% |
| ## | LM_comp | \| | 0.13 | \| | [-0.03, | 0.31] | \| | 94.30% |
| ## | Braak1 | \| | -9.36e-03 | \| | [-0.70, | 0.51] | \| | 51.60% |
| ## | anxdep_comp | \| | -0.19 | \| | [-0.45, | -0.02] | \| | 99.20% |
| ## | ERC_bilateral_sum_adj | \| | -0.31 | \| | [-1.07, | 0.46] | \| | 79.80% |
| ## | Age | \| | -0.05 | \| | [-0.16, | 0.06] | \| | 83.10% |
| ## | sexCode | \| | 1.02 | \| | [-0.25, | 2.18] | \| | 94.40% |
| ## | Edu | \| | 0.06 | \| | [-0.11, | 0.21] | \| | 74.40% |
| ## | Braak1:anxdep_comp | \| | -0.03 | \| | [-0.44, | 0.23] | \| | 60.80% |

m.metamem.tau.braak2 <- lm(data = d.cog.meta.tau, metamem_comp ~ LM_comp + Braak2*anxdep_comp + HC_bilatera l_sum_adj + Age + sexCode + Edu)

check_model(m.metamem.tau.braak2)


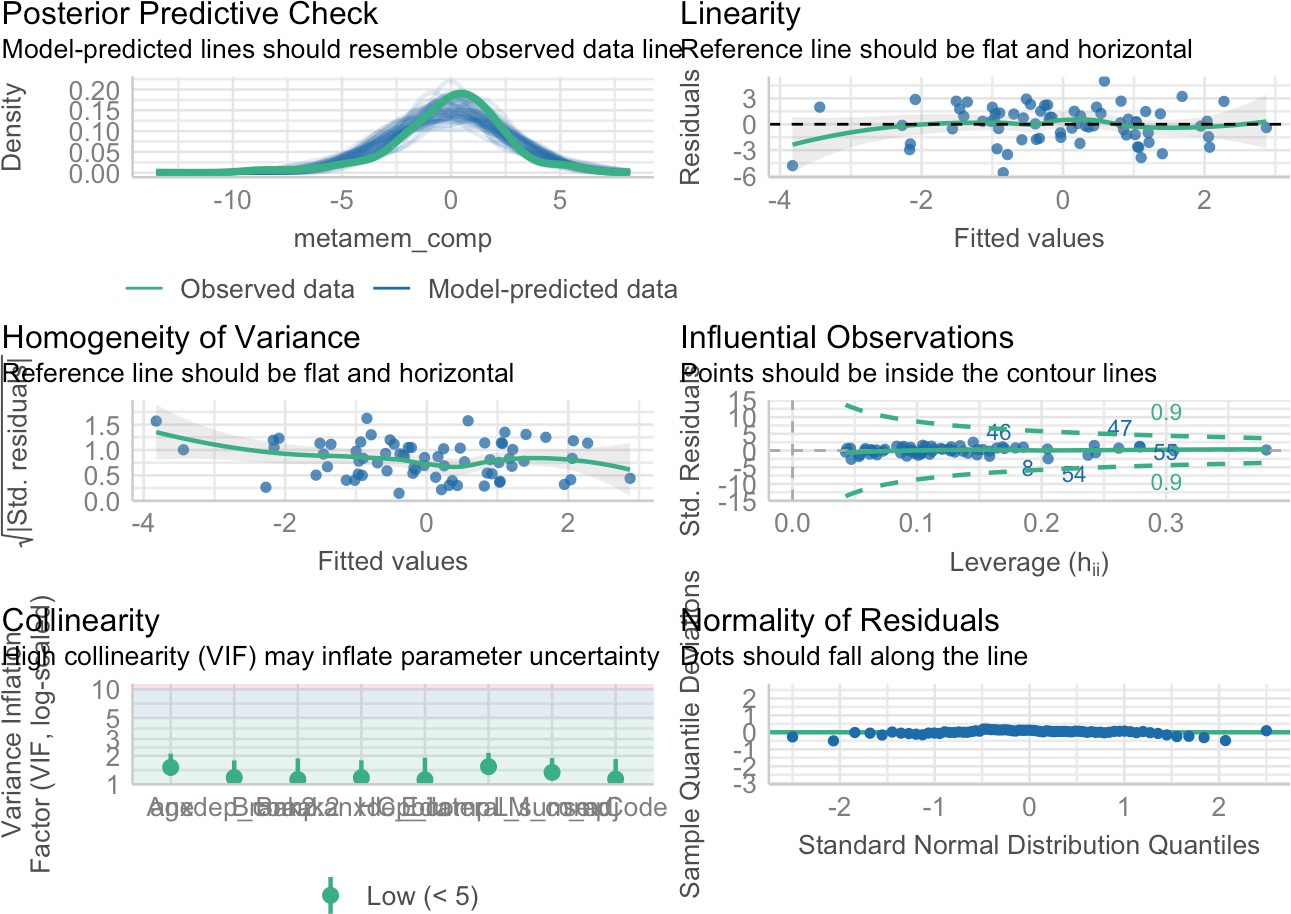


bootstrap_parameters(m.metamem.tau.braak2, test = "pd")

| ## | # Fixed Effects |  |  |  |  |  |  |  |
| --- | --- | --- | --- | --- | --- | --- | --- | --- |
| ## |  |  |  |  |  |  |  |  |
| ## | Parameter | \| | Coefficient | \| |  | 95% CI | \| | pd |
| ## |  |  |  |  |  |  |  |  |
| ## | (Intercept) | \| | 1.72 | \| | [-6.22, | 10.76] | \| | 65.50% |
| ## | LM_comp | \| | 0.21 | \| | [ 0.05, | 0.39] | \| | 99.40% |
| ## | Braak2 | \| | -0.12 | \| | [-0.65, | 0.36] | \| | 68.00% |
| ## | anxdep_comp | \| | -0.21 | \| | [-0.44, | -0.05] | \| | 99.90% |
| ## | HC_bilateral_sum_adj | \| | -0.42 | \| | [-1.15, | 0.27] | \| | 87.30% |
| ## | Age | \| | -0.05 | \| | [-0.18, | 0.07] | \| | 77.30% |
| ## | sexCode | \| | 1.18 | \| | [ 0.14, | 2.25] | \| | 99.10% |
| ## | Edu | \| | 0.05 | \| | [-0.10, | 0.20] | \| | 78.00% |
| ## | Braak2:anxdep_comp | \| | -0.25 | \| | [-0.46, | -0.03] | \| | 98.50% |
